# A deficient CP24 allele defines variation for dynamic nonphotochemical quenching and photosystem II efficiency in maize

**DOI:** 10.1093/plcell/koaf063

**Published:** 2025-03-25

**Authors:** John N Ferguson, Leonardo Caproni, Julia Walter, Katie Shaw, Lucia Arce-Cubas, Alice Baines, Min Soe Thein, Svenja Mager, Georgia Taylor, Lee Cackett, Jyotirmaya Mathan, Richard L Vath, Leo Martin, Bernard Genty, Mario Enrico Pè, Tracy Lawson, Matteo Dell’Acqua, Johannes Kromdijk

**Affiliations:** Department of Plant Sciences, University of Cambridge, Cambridge, Cambridgeshire CB2 3EA, UK; School of Life Sciences, University of Essex, Wivenhoe Park, Colchester, Essex CO4 3SQ, UK; Institute of Plant Sciences, Scuola Superiore Sant’Anna, 56127 Pisa, Italy; Department of Plant Sciences, University of Cambridge, Cambridge, Cambridgeshire CB2 3EA, UK; Department of Plant Sciences, University of Cambridge, Cambridge, Cambridgeshire CB2 3EA, UK; Department of Plant Sciences, University of Cambridge, Cambridge, Cambridgeshire CB2 3EA, UK; Department of Plant Sciences, University of Cambridge, Cambridge, Cambridgeshire CB2 3EA, UK; Institute of Plant Sciences, Scuola Superiore Sant’Anna, 56127 Pisa, Italy; Institute of Plant Sciences, Scuola Superiore Sant’Anna, 56127 Pisa, Italy; Department of Plant Sciences, University of Cambridge, Cambridge, Cambridgeshire CB2 3EA, UK; Department of Plant Sciences, University of Cambridge, Cambridge, Cambridgeshire CB2 3EA, UK; School of Life Sciences, University of Essex, Wivenhoe Park, Colchester, Essex CO4 3SQ, UK; Department of Plant Sciences, University of Cambridge, Cambridge, Cambridgeshire CB2 3EA, UK; Commissariat à l'Energie Atomique, Centre National de la Recherche Scientifique, Unité Mixte de Recherche 7265, Institut de Biosciences et Biotechnologies Aix-Marseille, Université Aix-Marseille, Saint-Paul-lez-Durance 13108, France; Commissariat à l'Energie Atomique, Centre National de la Recherche Scientifique, Unité Mixte de Recherche 7265, Institut de Biosciences et Biotechnologies Aix-Marseille, Université Aix-Marseille, Saint-Paul-lez-Durance 13108, France; Institute of Plant Sciences, Scuola Superiore Sant’Anna, 56127 Pisa, Italy; School of Life Sciences, University of Essex, Wivenhoe Park, Colchester, Essex CO4 3SQ, UK; Institute of Plant Sciences, Scuola Superiore Sant’Anna, 56127 Pisa, Italy; Department of Plant Sciences, University of Cambridge, Cambridge, Cambridgeshire CB2 3EA, UK; Institute for Genomic Biology, University of Illinois at Urbana-Champaign, Urbana, IL 61801, USA

## Abstract

Maize (*Zea mays* L.) is a global crop species in which CO_2_ assimilation occurs via the C_4_ pathway. C_4_ photosynthesis is typically more efficient than C_3_ photosynthesis under warm and dry conditions; however, despite this inherent advantage, considerable variation remains in photosynthetic efficiency for C_4_ species that could be leveraged to benefit crop performance. Here, we investigate the genetic architecture of nonphotochemical quenching (NPQ) and photosystem II (PSII) efficiency using a combination of high-throughput phenotyping and quantitative trait loci (QTL) mapping in a field-grown Multi-parent Advanced Generation Inter-Cross (MAGIC) mapping population. QTL mapping was followed by the identification of putative candidate genes using a combination of genomics, transcriptomics, protein biochemistry, and targeted physiological phenotyping. We identified four genes with a putative causal role in the observed QTL effects. The highest confidence causal gene was found for a large effect QTL for photosynthetic efficiency on chromosome 10, which was underpinned by allelic variation in the expression of the minor PSII antenna protein light harvesting complex photosystem II subunit (LHCB6 or CP24), mainly driven by poor expression associated with the haplotype of the F7 founder line. The historical role of this line in breeding for early flowering time may suggest that the presence of this deficient allele could be enriched in temperate maize germplasm. These findings advance our understanding of the genetic basis of NPQ and PSII efficiency in C_4_ plants and highlight the potential for breeding strategies aimed at optimizing photosynthetic efficiency in maize.

## Teaser

High throughput phenotyping of a maize multi-founder mapping population reveals genetic factors controlling dynamic photoprotection and photosystem II efficiency in maize.

## Introduction

Maize (*Zea mays* L.) is one of the most important cereal crops in the world and is widely cultivated for food, feed, and fuel (Haarhoff and Swanepoel 2020). Current maize yield trends, when projected in future climate scenarios, may be insufficient to meet production demands and require new approaches to improve productivity in a sustainable way (Ray et al. 2013; Eckardt et al. 2023). Photosynthesis, the process by which solar energy is converted to chemical energy, is the primary source of plant productivity. Previous work has suggested that targeted manipulation of photosynthetic pathways can contribute to resilience and yield potential in maize (e.g. Salesse-Smith et al. 2018, 2020; Wu et al. 2019) and other species (e.g. Yoon et al. 2020; De Souza et al. 2022), making it a promising avenue to develop the crop varieties of the future (Ort et al. 2015).

Maize uses C_4_ photosynthesis, which under warmer and drier environments is more efficient than C_3_ photosynthesis due to a molecular pump that concentrates CO_2_ close to the site of ribulose bisphosphate (RuBP) carboxylase-oxygenase (Rubisco) expression, consequently suppressing RuBP oxygenase activity and associated energetic losses via the photorespiration pathway (Hatch 1987). Despite the inherent advantage of the C_4_ photosynthetic pathway over the C_3_ pathway, there is still margin to improve photosynthetic efficiency in maize, which could in turn result in increased productivity (Salesse-Smith et al. 2018) and contribute to mitigate environmental stress (Doron et al. 2020; Salesse-Smith et al. 2020). Increasing photosynthetic efficiency could therefore be an important trait to maintain current and future productivity of maize and other C_4_ crops (see also review by Sales et al. (2021)).

One way to improve photosynthesis is by improving the capacity of the plant to effectively use incoming photons. Excessive light energy absorbed by the photosynthetic antennae enhances the probability of formation of reactive oxygen species (ROS), which can inflict oxidative damage to thylakoid components and thereby lead to deactivation of photochemistry (Aro et al. 1993). Photoinhibition gives rise to sustained downregulation of photosynthetic efficiency and can therefore reduce crop carbon gain and associated growth and productivity (Long et al. 1994). To reduce the risk of photoinhibition, photoprotective nonphotochemical quenching (NPQ) is induced under high light, which dissipates excess energy in a controlled manner (Murchie and Ruban 2020). The sporadic nature of light availability in crop canopies (Long et al. 2022) means that rapid induction and relaxation responses of NPQ are necessary to adequately adjust the efficiency of light-harvesting to the intensity of intercepted light.

Model simulations (Zhu et al. 2013) and proof of concept studies in field-grown tobacco (*Nicotiana tabacum*) and soybean (*Glycine max*) (Kromdijk et al. 2016; De Souza et al. 2022) suggest that accelerating NPQ relaxation can enhance crop yields by reducing the lag time to return to high PSII operating efficiency and CO_2_ fixation upon a switch from high to low light. In addition, enhancing the NPQ amplitude and rate of induction have also been linked with increased yields in rice (Hubbart et al. 2018). These pioneering proof-of-concept studies have been conducted on C_3_ crops, focusing on known NPQ determinants such as the PSII subunit S (PSBS), violaxanthin de-epoxidase (VDE) and zeaxanthin epoxidase (ZEP). Only relatively few studies have explored NPQ kinetics in species that perform photosynthesis via the C_4_ pathway, which may alter NPQ responses (Guidi et al. 2019). Genetic factors involved in NPQ determination can be identified using forward genetics approaches on diversity panels, as recently reported in maize (Sahay et al. 2023) and sorghum (*Sorghum bicolor*) (Sahay et al. 2024), who characterized the genetic determinants of variation in NPQ kinetics using collections of lines grown in the US Midwest, Nebraska. These studies identified multiple, independent candidate genes underlying NPQ variation, whose roles in photoprotection were validated using mutants of *Arabidopsis thaliana* (Arabidopsis) putatively orthologous genes.

In our study, we aimed to expand upon the work of Sahay et al. (2023) by phenotyping dynamic photoprotection and PSII efficiency of field-grown maize close to the northern latitudinal range limit for maize cultivation, where cold acclimatation could be achieved through optimized photoprotection (reviewed by: Burnett and Kromdijk 2022). Additionally, we focused on elucidating the genetic underpinning of these traits using a Multiple parent Advanced Generation Inter-Cross (MAGIC) maize population (Dell’Acqua et al. 2015), thereby enabling high-power and high-resolution genetic mapping. Maize segregating populations, like the MAGIC panel used in our study and the Nested Association Mapping (NAM) maize panel (Yu et al. 2008), have been utilized to characterize and study the architecture of numerous complex phenotypes (Gage et al. 2020; Scott et al. 2020) and, given their nature, represent invaluable community assets for genetic studies in maize. In this study, we detected substantial and consistent variation for NPQ and PSII efficiency and their responses to dynamic irradiance which allowed for the identification of 42 associated QTL. From these QTL, we used genomics, transcriptomics, protein biochemistry and targeted physiological phenotyping to provide further evidence to support the role of four genes in the regulation of NPQ and PSII photochemistry. Of these four, we particularly highlight a role for a deficient allele of the CP24 minor photosystem II antenna protein which may be more prevalent in temperate germplasm and has a strong negative impact on photosynthetic efficiency.

## Results

### Genetic variation exists for PSII efficiency and NPQ across the MAGIC maize population

**Figure 1.**
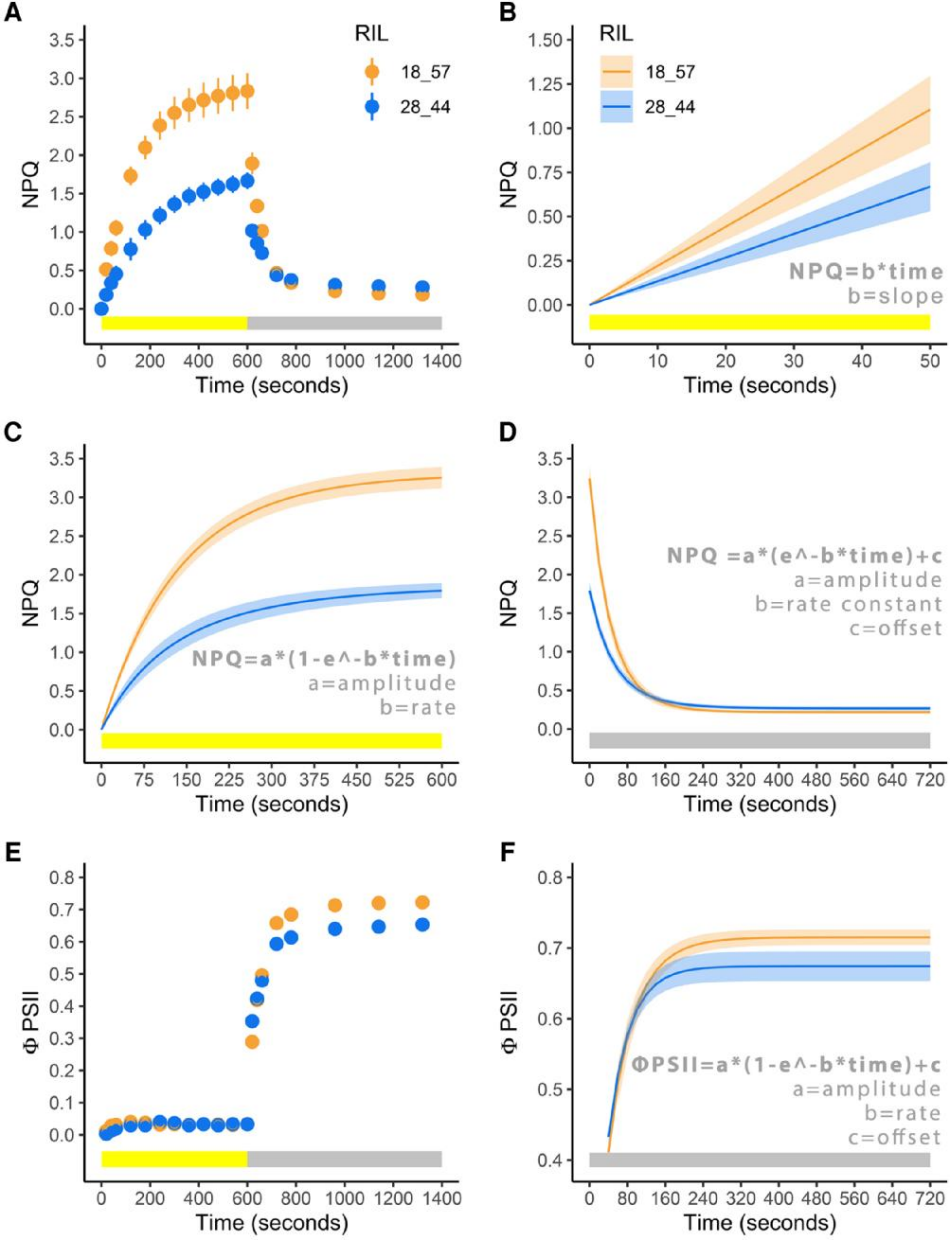
Example of models used to fit the dynamic NPQ and operating PSII efficiency (ΦPSII) data based on 2 distinct RILs (SSA_00157 and SSA_00376). Data shown are from *N* = 6 biological replicates of each RIL. **A)** NPQ throughout the measurement protocol. Circles represent the mean value, and the bars represent the standard error of the mean. **B)** Initial NPQ induction modeled via a linear regression. The solid line represents the mean of the predicted NPQ according to the linear regression fit and the ribbon represents the standard error of the predicted fit. **C)** NPQ induction modeled via an exponential equation. **D)** NPQ relaxation modeled via an exponential model. **E)** ΦPSII through the measurement protocol. **F)** ΦPSII recovery modeled via an exponential model.

**Figure 2.**
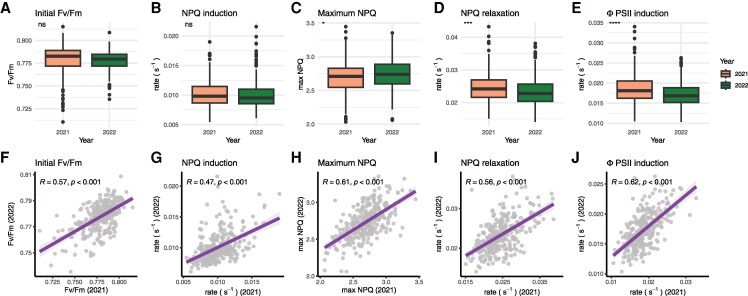
Comparison of selected parameters across the 2 experimental years. **A to E)** Boxplots demonstrating population wide variation across both experimental years for maximum efficiency of photosystem II (*F*_v_/*F*_m_), NPQ induction rate, maximum NPQ, NPQ relaxation rate, and operating PSII efficiency(ΦPSII) recovery rate. The boxes of the boxplots denote the median and interquartile range. The whiskers show the minimum and maximum range, with RILs falling away from that range being shown as individual circles (2021: *N* = 316; 2022: *N* = 312). **F to H)** Scatter plots demonstrating associations between the same traits across each experimental year. Correlations were statistically tested via linear models, with the associated regression line and standard error being denoted as the fit and associated shaded area respectively (*N* = 301). Significant differences and correlations are denoted at the following *P*-value levels: * 0.05, ** 0.01, *** 0.001, **** 0.0001, where ns, nonsignificant (i.e. *P* > 0.05).

To discover and map allelic variation for maximum PSII efficiency (Fv/Fm), operating PSII efficiency (ΦPSII) and NPQ as well as their responses to dynamic irradiance, 320 MAGIC maize recombinant inbred lines (RILs) were grown in a replicated alpha-lattice design in the east of the United Kingdom (52.2 °N, 0.1 °E) for 2 consecutive field seasons (2021 and 2022). We successfully phenotyped 315 RILs in 2021 and 312 in 2022, where 301 RILs were common between the 2 yr (Supplementary Tables S1 and S2). This represented data collected from more than 3700 leaves in total. Chlorophyll fluorescence imaging of leaf segments was employed to determine timeseries data for photosystem II efficiency (ΦPSII) and NPQ induction and relaxation in response to 10 min high light exposure followed by 12 min dark recovery. We have previously confirmed that measuring these traits on detached leaves for the benefit of high throughput does not introduce bias (Ferguson et al. 2023). The resulting NPQ and ΦPSII values were analyzed separately for each measured timepoint as well as modeled across the light and dark phases of the protocol (Fig. 1). Substantial genetic variation was detected for all measured and modeled traits in both years (Fig. 2; Supplementary Fig. S1 and Tables S1 and S2). The 2022 growing season was drier and warmer than the 2021 growing season and necessitated irrigation (Supplementary Fig. S2). Consistent with the drier and warmer conditions in 2022, the population average for maximum NPQ was higher and the rate constants of relaxation of NPQ and recovery of ΦPSII following the actinic light being switched off were slower than in 2021 (Fig. 2). Despite these environmental differences, the population-wide range of variation was similar across both experimental years and strong year-by-year correlations were found (Fig. 2; Supplementary Fig. S1). This was reflected in broad-sense heritability across all traits ($H_{B}^{2}$), computed both for each year individually and jointly, ranging between 0.3 to 0.7 (Supplementary Figure S3).

Pair-wise trait correlations were largely conserved across the 2 yr or when testing using predicted means derived from both years. Indeed, 75 (out of a possible 91) significant trait interactions held true between 2021 and 2022 (Supplementary Table S3). Significant negative associations between the rate constants of NPQ induction and relaxation were observed across both years, suggesting that RILs with fast rates of induction of NPQ had slower rates of relaxation and vice versa. Since the NPQ relaxation rate constant positively correlated with the rate constant of recovery of ΦPSII, the latter was also observed to have a significant negative correlation with the rate constant of NPQ induction across both years (Supplementary Table S3).

### Multiple QTL control ΦPSII and NPQ and with varying effects depending on the light environment

**Figure 3.**
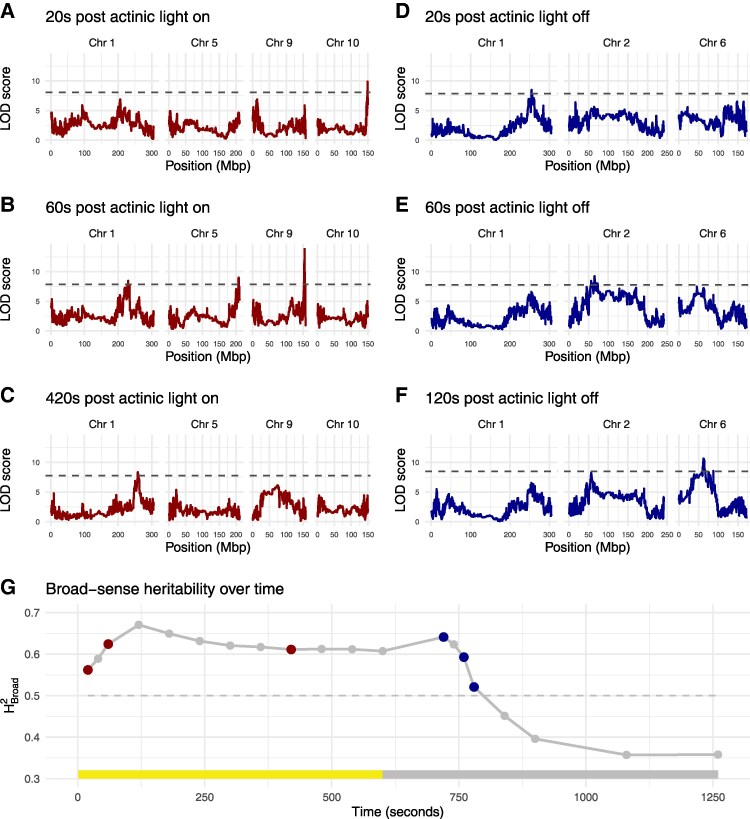
QTL mapping of NPQ at different timepoints within the induction (light) and relaxation (dark) phases. Mapping is performed using the predicted means derived from the joint-year linear mixed effect models. **A)** QTL for NPQ 20 s after the actinic light is switched on. **B)** QTL for NPQ 60 s after the actinic light is switched on. **C)** QTL for NPQ 420 s after the actinic light is switched on. **D)** QTL for NPQ 20 s after the actinic light is switched off. **E)** QTL for NPQ 60 s after the actinic light is switched off. **F)** QTL for NPQ 120 s after the actinic light is switched off. **G)** Broad-sense heritability ($H_{B}^{2}$) for NPQ over time.

MAGIC maize RILs and founder lines were genotyped with 74,706 single nucleotide polymorphism (SNP) markers. These were used to probabilistically reconstruct the RIL genomes according to haplotypes of founder lines. We first focused on the identification of QTL for NPQ and ΦPSII at each timepoint throughout the measurement protocol, based on predicted means derived from the joint year model. The QTL mapping results for these traits showed that the genomic regions associated with the observed variation were dependent on the time elapsed following the actinic light being switched on or off (Supplementary Videos S1 to S4). For example, variation in NPQ during the light induction phase of the experimental protocol was initially defined by a QTL on chromosome 10 (Fig. 3A) which rapidly disappeared before QTL on chromosomes 9, 5, and 1 sequentially appeared (Fig. 3, B and C). Similarly, variation in NPQ during the dark relaxation phase was initially associated with the same chromosome 1 QTL as the light phase (Fig. 3D), before other QTL on chromosomes 2 (Fig. 3E) and 6 (Fig. 3F) appeared. The genetic architecture of ΦPSII in the MAGIC panel was relatively less complex. A highly significant large-effect QTL for dark-adapted ΦPSII (*F_v_/F_m_*) was identified on chromosome 10 and co-located with the QTL for induction phase NPQ (shown in Fig. 3A). This QTL was largely present throughout both the light phase (where ΦPSII declines, Fig. 1D) and during the dark phase (where ΦPSII recovers, Fig. 1F) where it gradually increased in significance (Supplementary Fig. S4, C and D and Videos S3 and S4). An additional QTL for ΦPSII during the light phase was found on chromosome 9 for time-points from 60 s onwards (Supplementary Fig. S4B).

The same QTL were also found when mapping the fluorescence parameters that were determined from combined analysis of several time-points, representing the time series of the parameter. The QTL on chromosome 10 was also detected for the photoprotection index (PI) and for the y-axis intercept from the exponential model describing the recovery of ΦPSII. PI is a modification of the pNPQ approach outlined by Ruban and Murchie (2012), which provides a measure of the “photoprotectiveness” of NPQ following high light exposure by accounting for the predicted decrease in PSII efficiency due to NPQ. To identify pleiotropic QTL such as the Chr 10 QTL, which affect several traits in parallel, is not surprising and reflects the high correlation existing between these traits and their common association with PSII photochemistry (Supplementary Table S3). The rate constant of NPQ induction was estimated by the initial slope of NPQ during the first 60 s of the light phase as well as by a first-order exponential model for the whole light phase, both of which were associated with the QTL on chromosome 9 previously found for several early timepoints during the light phase (Tables 1 and 2). The QTL on chromosome 1 found later in the light phase and early dark phase, was also found for the amplitude of NPQ induction, amplitude of NPQ relaxation and amplitude of ΦPSII recovery. This is consistent with the mechanistic links between NPQ relaxation and recovery of ΦPSII following a change in light intensity (Table 2).

**Table 1.** QTL associated with NPQ and ΦPSII at each measured timepoint

Period	Model	Phenotype	Chr.	Average peak position (Mbp)	Max LOD	Min CI- low (Mbp)	max CI- high (Mbp)	Time (s)
Dark	Joint-year	NPQ	1	255	8.5	247.0	262.1	20
Dark	Joint-year	phiPSII	10	149.2	19.9	147.8	150	20, 40, 60, 120, 180, 360, 540
Light	Joint-year	NPQ	10	148.7	9.9	147.7	149.5	20
Light	Joint-year	NPQ	9	154.4	15.6	152.9	154.5	40, 60, 120
Light	Joint-year	NPQ	5	208.1	9.0	199.5	209.7	60, 120
Light	Joint-year	NPQ	1	258.4	8.5	248.3	263.5	240, 300, 360, 420, 480, 540, 600
Light	Joint-year	NPQ	1	229.8	8.5	204.5	234.3	60
Light	Joint-year	phiPSII	10	149.1	21.3	147.8	149.5	20, 40, 60, 180, 240, 300, 360, 420, 480, 540, 600
Light	Joint-year	phiPSII	9	154.4	11.6	154.0	155.4	60, 120
Dark	2021	phiPSII	10	149.2	19.0	147.7	150.0	20, 40, 60, 120, 180, 360, 540
Light	2021	NPQ	10	148.7	11.3	146.3	149.0	480
Light	2021	NPQ	9	154.4	13.7	152.0	154.7	40, 60, 120
Light	2021	NPQ	5	207.8	10.9	195.5	210.0	60, 120
Light	2021	NPQ	3	158.6	8.5	150.3	164.5	240, 300, 360, 420, 480, 540, 600
Light	2021	phiPSII	10	149.0	20.6	148.5	149.5	20, 40, 60, 240, 300, 360, 420, 480, 540, 600
Light	2021	phiPSII	9	154.4	10.7	154.0	155.6	60, 120
								
Dark	2022	NPQ	2	240.6	10.4	240.3	241.9	180
Dark	2022	NPQ	1	249.3	10.8	215.1	260.9	120, 180
Dark	2022	NPQ	1	229.8	8.6	215.1	234.0	60
Dark	2022	phiPSII	10	149.2	12.6	148.0	149.5	120, 180, 360, 540
Light	2022	NPQ	9	154.4	11.8	136.3	155.2	60, 120, 180
Light	2022	phiPSII	10	149.2	12.6	148.0	150.0	20, 50, 480, 540, 600
Light	2022	phiPSII	9	154.5	10.6	154.4	155.3	120, 180

**Table 2.** QTL associated with *F*_v_/*F*_m_ and traits derived from the linear and exponential modeling of NPQ induction, NPQ relaxation and ΦPSII recovery

Phenotype	Model	Chromosome	Peak position (Mbp)	LOD value	CI- Low (Mbp)	CI- high (Mbp)	QTL span (Mbp)
Fv/Fm		10	149.2	21.8	149.0	149.2	0.2
NPQ induction amplitude	Joint-Year	1	258.2	7.7	248.8	263.1	14.3
NPQ induction linear	Joint-Year	9	154.4	10.1	152.9	155.2	2.3
NPQ induction rate	Joint-Year	9	154.4	15.7	154.3	154.4	0.1
NPQ relaxation amplitude	Joint-Year	1	258.4	8.4	248.3	263.0	14.8
phiPSII induction amplitude	Joint-Year	1	258.4	8.1	247.4	263.0	15.6
phiPSII induction residual	Joint-Year	10	149.0	12.2	148.7	149.5	0.8
PI	Joint-Year	10	149.2	19.4	148.9	149.2	0.3
Fv/Fm	2021	10	149.2	20.3	148.9	149.4	0.4
NPQ induction rate	2021	5	207.1	8.3	199.5	210.1	10.6
NPQ induction rate	2021	9	154.4	16.7	154.1	154.4	0.3
NPQ relaxation amplitude	2021	3	158.6	7.9	151.0	163.4	12.4
phiPSII induction amplitude	2021	3	158.6	7.9	151.5	162.4	10.9
phiPSII induction rate	2021	6	43.8	9.4	38.7	64.6	25.9
phiPSII induction residual	2021	10	149.0	11.0	148.7	149.5	0.8
PI	2021	10	149.2	18.8	148.9	149.4	0.4
							
Fv/Fm	2022	10	149.2	12.2	148.8	149.2	0.4
phiPSII induction amplitude	2022	5	22.1	8.2	21.2	23.3	2.1
PI	2022	10	149.2	10.3	148.7	149.2	0.5

### A multi-faceted approach for identification of candidate genes underpinning QTL

Altogether, the QTL identified contained 3,064 unique gene models (Supplementary Table S4). For all QTL-associated gene models we identified the Arabidopsis gene with the highest sequence similarity and used gene ontology to find genes with known roles in photosynthesis and in the detoxification of ROS (summarized in Supplementary Fig. S5). However, exclusive prioritization based on gene ontology annotations would rule out discovery of genes with no prior association to the phenotypes measured here. We therefore also used 2 alternative methods based on genomics to prioritize candidate genes within confidence intervals of QTL (Dell’Acqua et al. 2015). The first method considers the level of expression of gene models in the QTL interval as measured on the founder lines of the MAGIC population and evaluates correlations with the founder haplotype effects, i.e. coefficients, estimated at the QTL locus. The underlying hypothesis is that the contribution of a founder haplotype at the QTL may be driven by constitutive differences in gene expression at the locus. The second method uses SNP markers derived from the full genome sequence available for the founder lines, imputing SNP alleles on reconstructed haplotypes in the region of the QTL with the aim of identifying gene models with variants associated with the variation of phenotypes via a local GWAS. These are both indirect approaches based on assumptions that may or may not manifest in different QTL, i.e. the phenotype may be due to the difference in expression level or differences in sequences, by neither, or by both. Hence, the 2 methods are used in combination, and provide partial results. Gene models whose expression level in the founder lines was significantly associated with founder coefficients at QTL are reported in Supplementary Table S5. Gene models associated with imputed SNP variants in QTL mapping intervals are reported in Supplementary Table S6. The following 2 sections detail the further exploration of these results, by corroborating candidate genes identified with these 2 methods by means of analyzing Arabidopsis mutants for the genes with the highest sequence similarity, as well as additional molecular, biochemical, and physiological measurements on distinct founder lines.

### Correlating transcript abundance variation with founder haplotype effects identifies candidate genes for NPQ regulation

**Figure 4.**
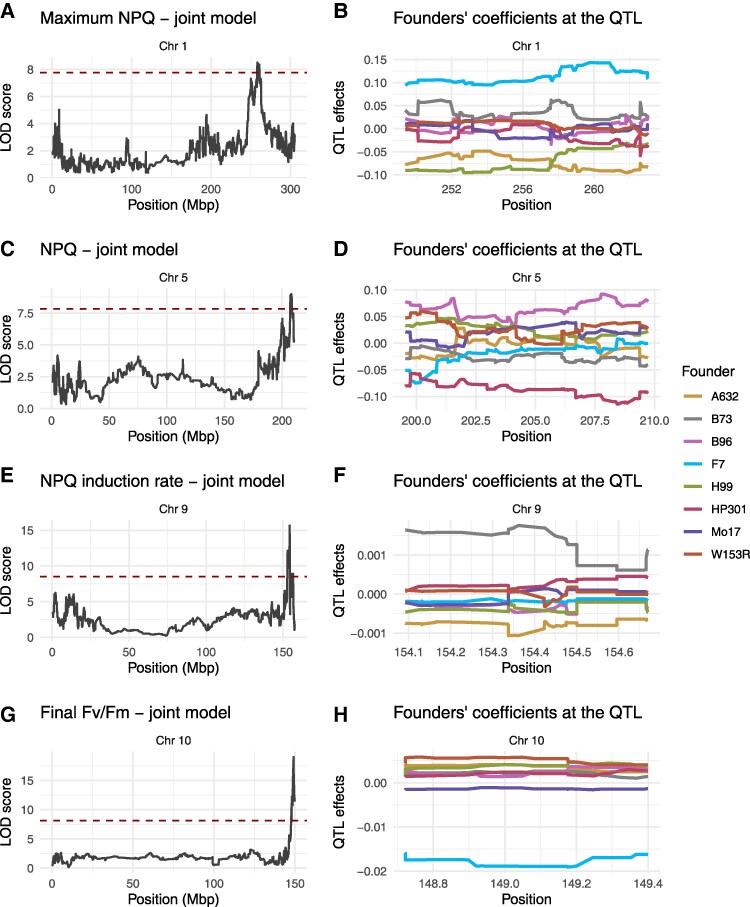
Key QTL identified for NPQ and *F*_v_/*F*_m_. **A, C, E, G)** Main effect QTL for selected traits; **B, D, F, H)** Founder effects associated with each QTL. NPQ, non-photochemical quenching; *F*_v_/*F*_m_, maximum efficiency of photosystem II.

For each of the joint year QTL on chromosomes 1, 5, 9, and 10 (Fig. 4, A, C, E, and G) we determined the contribution of different founder haplotypes as reconstructed by the mapping procedure (Fig. 4, B, D, F, and H). The same founder haplotype may contribute with different effects in different QTL; for example, the founder line F7 was contributing with a positive effect in the QTL on chromosome 1, and with a markedly negative effect in the QTL on chromosome 10. We correlated transcript abundance of all genes within these QTL intervals as measured by RNA sequencing with said founder contributions. This approach identified 3 genes within the QTL on chromosome 1, 3 within the QTL on chromosome 5, and 4 within the QTL on chromosome 10, while none were found for the QTL on chromosome 9. We phenotyped T-DNA insertional mutants of the Arabidopsis orthologs of these genes to further test their potential roles as described below.

The Arabidopsis genes with the highest sequence similarity to the 3 genes within the QTL on chromosome one were a protein involved in cell division regulation (CDC48) and an uncharacterized transducing repeat protein (AT5G02430). The remaining gene within this QTL and identified through this expression association approach was orthologous to 2 reversibly glycosylated polypeptides (RGP1 and RGP2). Arabidopsis mutants for *cdc48* and for the transducing repeat protein (SALK_136212C) did not show significantly different NPQ values relative to wild type during the induction and relaxation phases (Supplementary Fig. S6, B and C). The *rgp2* mutant, however, demonstrated significantly enhanced NPQ relative to wild type during the end of the induction phase (Supplementary Fig. S6A). This mirrors the identification of this QTL for maximum NPQ (as well as NPQ at other points (Fig. 4, Table 1) and may suggest a role for reversible glycosylated polypeptides in NPQ regulation.

The Arabidopsis genes with the highest sequence similarity to the 3 genes within the QTL on chromosome 5 were a gamma-tocopherol methyltransferase gene (*VTE4*; AT1G64970), an uncharacterized phosphofructokinase family protein (AT1G76550), and an uncharacterized ARID-domain containing protein (AT2G17410). We observed no difference in NPQ relative to wild type for *vte4* or the mutant of the ARID-domain containing protein (SALK_026835C; Supplementary Fig. S7, B and C). However, NPQ was significantly reduced in the mutant of the phosphofructokinase family protein (SAIL_1151_H05) at 8 points during induction and relaxation (Supplementary Fig. S7A), which may suggest a role for this gene in NPQ regulation and agrees with the traits with which the associated QTL was identified (Fig. 4C, Table 1).

The Arabidopsis genes with the highest sequence similarity to the 4 genes within the QTL on chromosome 10 included the minor PSII antenna protein (*CP24*), an uncharacterized clathrin light chain protein (AT2G20760), and an uncharacterized aluminum-induced protein (AT4G27450). The final associated gene was orthologous to 2 Arabidopsis K + efflux antiporter proteins (KEA1 and KEA2, Supplementary Fig. S8A). The role of *CP24* for photosynthetic electron transport is well characterized in Arabidopsis, and mutants have significantly reduced *F*_v_/*F*_m_ (Kovacs et al., 2006; de Bianchi et al. 2008) in line with the QTL effect observed here. *KEA1* and *KEA2* are part of an extended K^+^/H^+^ antiporter family with distinct roles for specific isoforms which are conserved between Arabidopsis and maize (Kong et al. 2021). Whereas *KEA3* localizes to the thylakoid where the antiport activity directly affects lumen pH and thereby NPQ and ΦPSII (Armbruster et al. 2014), *KEA1* and *KEA2* localize to the chloroplast inner envelope (Ferro et al. 2010; Kunz et al. 2014), are involved in osmoregulation and chloroplast development (Aranda Sicilia et al. 2016) and do not directly affect thylakoid lumen pH (Aranda Sicilia et al. 2021) or *F_v_/F_m_* (Kunz et al. 2014) unless expression of both isoforms is disrupted simultaneously. Accordingly, *F*_v_/*F_m_* in Arabidopsis *kea1* and *kea2* knockout mutants was not different from the genetic background (Supplementary Fig. S9). Independent 3′ mRNA-seq data for the negative effect founder line (F7) and a neutral effect founder line (B73) from Cackett et al. (2023) were used to scrutinize expression levels for *ZmKEA1*, 2 and *3*. The moderate decrease in expression in F7 of *Zm00001d026645* (Supplementary Fig. S8C), the maize *AtKEA1/2* homolog located in the Chr 10 QTL, was mirrored by a moderate increase in F7 of transcripts of the other *ZmKEA1/2* isoform (*Zm00001d001788*, Supplementary Fig. S8B). In addition, *ZmKEA3* (*Zm00001d041308*) expression levels were invariant between both lines (Supplementary Fig. S8D). On this basis, it seems unlikely that expression level variation of *Zm00001d026645* underlies the Chr 10 QTL effect. Analysis of *F*_v_*/F*_m_ in Arabidopsis knockout mutant lines for AT4G27450 and AT2G20760 also did not observe any significant differences compared with wild type (Supplementary Fig. S9). Further testing of the role of *CP24* in the context of this QTL is described subsequently.

### A protein kinase is identified by local GWAS in the QTL interval for NPQ induction

**Figure 5.**
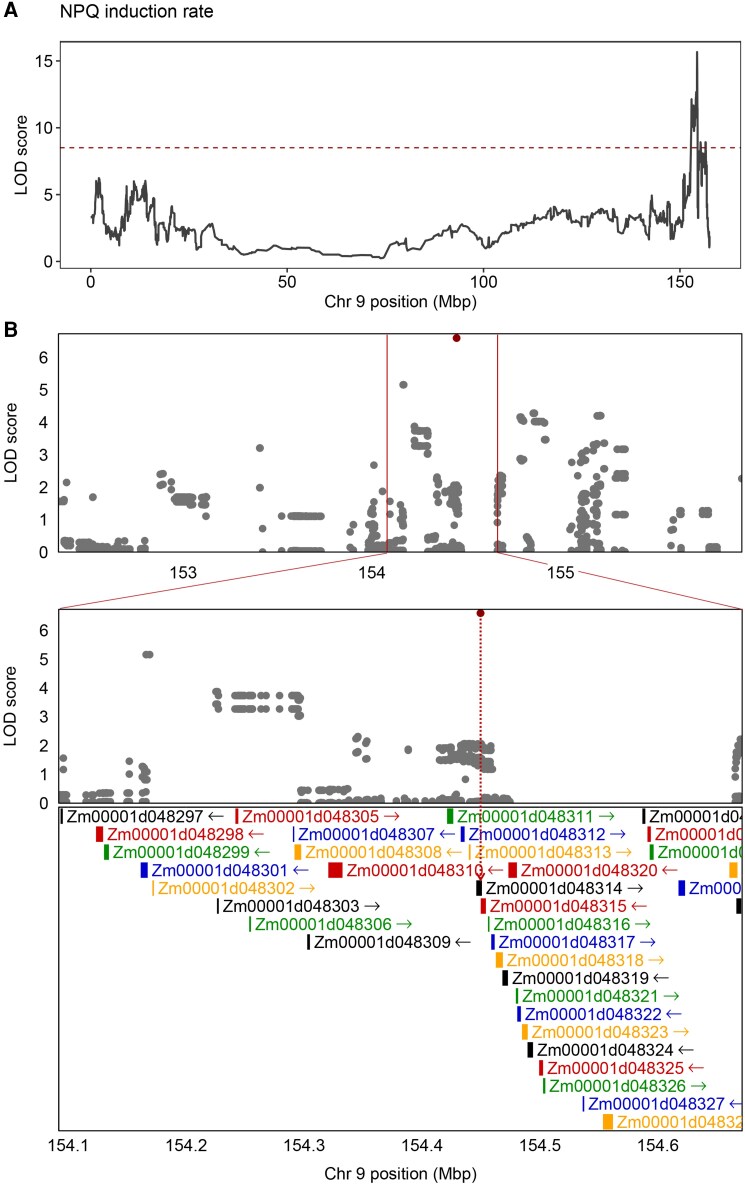
Identification of a putative chloroplastic protein kinase as a candidate gene regulating NPQ induction rate **A)** LOD score plot of NPQ induction rate on Chromosome 9 QTL, the horizontal dotted line represents the threshold of significance. **B)** top panel: SNP association mapping within a 4 Mbp region, centered on NPQ induction rate QTL on chromosome 9. Bottom panel: Zoomed in view of the same region (± 0.25 Mbp, centered on QTL), showing a putatively associated SNP falling within the coding region of *Zm0001d048314*. In this panel all 36 gene models annotated in this zoomed-in region are shown. In both panels, SNPs having LOD scores > 99^th^ percentile are highlighted in red.

Through our local GWAS approach (described above) we identified 23 significant trait-SNP associations within the confidence intervals of the most prominent QTLs (Fig. 3). Notably, only few of these associations were located within gene sequences (Supplementary Table S6). Interestingly, on the chromosome 9 QTL, for which we did not find any gene candidates based on correlated transcript levels, the local GWAS approach obtained a statistically significant signal within the sequence of *Zm0001d048314* (Fig. 5). This gene encodes a chloroplastic protein kinase according to PLAZA Integrative Orthology (Van Bel et al. 2018) matching an Arabidopsis orthologous gene family of protein kinases (Supplementary Table S7). These kinases include PROTEIN KINASE 1b (PK1B or APK1B) which has previously been implicated in light-induced stomatal opening (Elhaddad et al. 2014). We measured NPQ in Arabidopsis T-DNA insertional mutants of 3 Arabidopsis genes with high sequence similarity to *Zm0001d048314*, namely PK1B, CONSTITUTIVE DIFFERENTIAL GROWTH 1-LIKE 1 (CDL1) and APK1A. In all cases, these mutants showed significantly reduced NPQ at various points throughout the induction and relaxation phases (Supplementary Fig. S10, A to C) and the rate of NPQ induction was significantly reduced (Supplementary Fig. S10D) which is consistent with the trait for which this QTL was identified.

It is worth noting that the *carotenoid cleavage dioxygenase 1* (*CCD1*) gene was also found to be within the confidence interval of the chromosome 9 QTL. In maize, extensive *CCD1* copy number variation (CNV) has been documented and linked to enhanced carotenoid breakdown in white grain varieties (Tan et al. 2017). For completeness and due to the known role of carotenoids in ROS scavenging (Zakar et al. 2016), we checked for CNV in the founders using qPCR (Supplementary Table S8) and via WGS read counts (Supplementary Table S9). All major founders contained only 1 *CCD1* copy. CML91 was observed to have 10 copies, but since this contributes less than 5% of total allelic variation to the MAGIC population (Dell’Acqua et al. 2015), *CCD1* CNV seems very unlikely to underpin this QTL.

### A deficient CP24 allele explains the large effect QTL for NPQ and ΦPSII on chromosome 10

**Figure 6.**
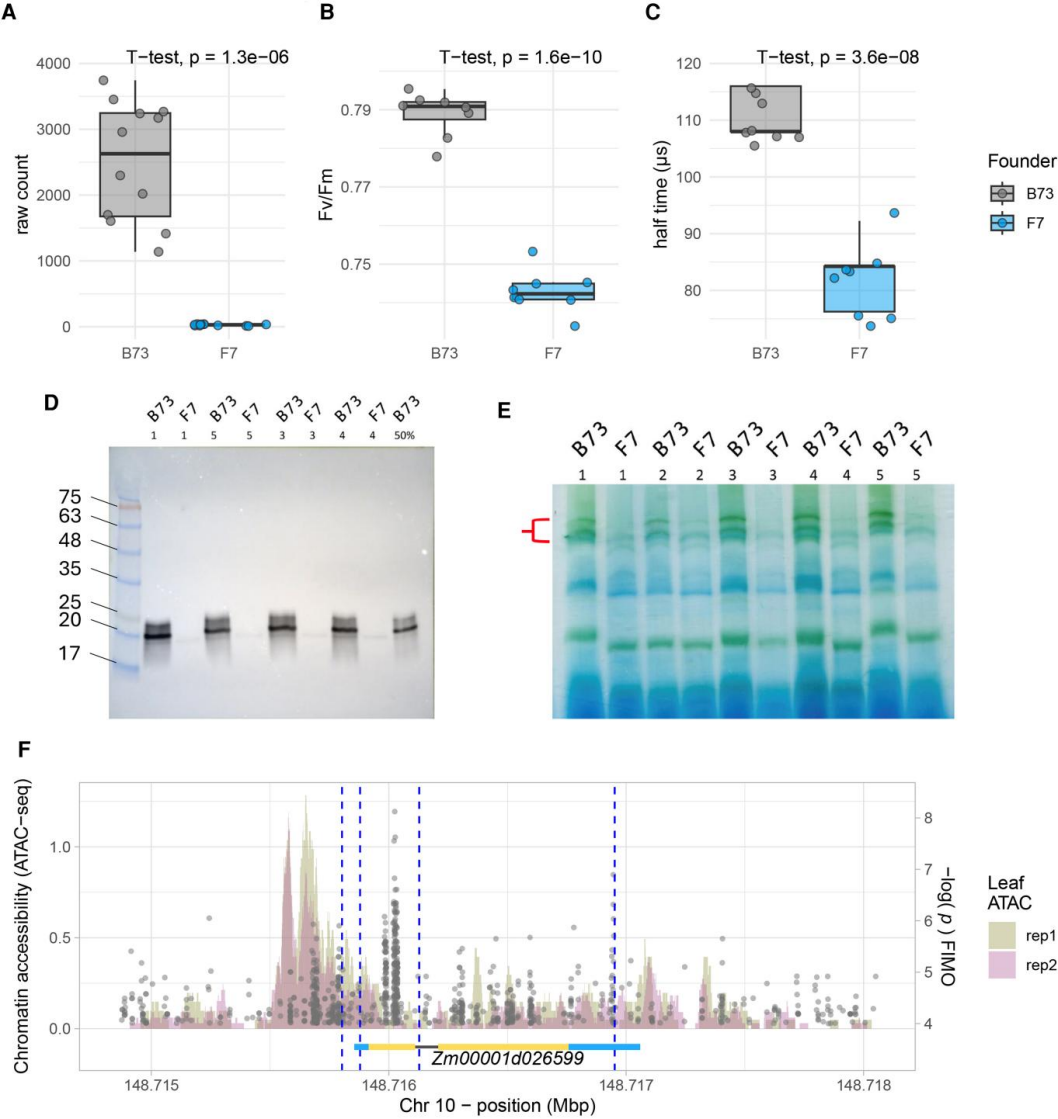
Differences in CP24-related phenotypes between B73 and F7 founder lines. **A)** Expression of CP24 (*t*-test, *P* = 1.3e^−6^, *N* = 12). **B)** Maximum efficiency of photosystem II (*F*_v_/*F*_m_) (*t*-test, *P* = 1.6e^−10^, *N* = 8) **C)** fluorescence rise time. (*t*-test, *P* = 3.6e^−8^, *N* = 8) **D)** Western blot of B73 and F7 thylakoid membrane protein samples using a CP24-specific antibody. **E)** Blue native gel electrophoresis (BN-PAGE) of thylakoid membrane complexes of B73 and F7. Red bracket indicates size of supercomplexes perturbed by absence of CP24 in F7 (F) Coding region analysis of the *CP24* gene (*Zm00001d026599*) ± 1000 bp. The barplot illustrates regions of accessible chromatin identified through ATAC-seq data generated from the fourth leaf by Ricci et al. (2019). Gray dots are positioned along the x-axis according to the physical position of predicted transcription factor binding site (TFBS) motifs relative to the B73 reference genome (V4; *y*-axis *P*-values represent the likelihood of a TFBS motif matching the sequence by random chance). Vertical blue dotted lines mark the positions of 4 unique variants within the F7 haplotype. The horizontal schematic shows the *CP24* gene structure: light blue segments represent the 5′ and 3′ untranslated regions (UTRs), yellow segments denote exons, and the black line indicates the intron.

We further explored a potential role of *CP24* in the large effect QTL on the distal portion of chromosome 10 which was found across many of the ΦPSII and NPQ parameters (Fig. 3; Supplementary Fig. S4 and Tables 1 and 2). For illustrative purposes, here we refer to data from the joint-year model for *F*_v_/*F*_m_ (Fig. 4G). The QTL effects estimated per founder haplotype clearly showed that this QTL was driven by a negative effect associated with the F7 haplotype (Fig. 4H). Independent 3′ mRNA-seq expression data for F7 and B73 (Cackett et al. 2023) confirmed significantly reduced *CP24* expression in F7 (Fig. 6A). The reduction in *F*_v_/*F*_m_ in F7 relative to B73 was also independently confirmed (Fig. 6B) as part of an in vivo assessment of fast fluorescence kinetics (Supplementary Fig. S11). Here, we observed significantly lower fluorescence rise times in F7 relative to B73 (Fig. 6C) and a lower *F*_v_/*F*_m_ mainly associated with an increase in minimum fluorescence (*F_o_*, Supplementary Fig. S11), both of which are similar to the alteration of light harvesting efficiency and photochemical yield observed previously in *cp24* mutant lines (Kovács et al. 2006; de Bianchi et al. 2008). Immunoblot analysis of B73 and F7 using a CP24-specific antibody confirmed that F7 also had severely reduced CP24 protein abundance compared with B73 (Fig. 6D). While the fainter band just below the strong CP24 band likely reflects some unspecific binding of the antibody, the band above the strong CP24 band has also been seen in previous studies using this antibody (Du et al. 2018; Myouga et al. 2018) and may reflect unprocessed CP24 protein. The reduced CP24 protein abundance in the F7 founder is also consistent with a decreased abundance of higher order PSII-LHCII supercomplexes in blue native PAGE analysis of purified thylakoid membranes from both founder lines (Fig. 6E) indicating a substantial truncation of PSII antenna size associated with the F7 haplotype. Altogether, these results are consistent with strong allelic variation in *CP24* expression driving the QTL effect on chromosome 10.

### The nature of the CP24 deficiency in F7 is inconclusive

To investigate a number of potential causes of poor *CP24* expression by the F7 allele, we utilized short-read whole-genome sequencing (WGS) data from the MAGIC maize founder lines (Dell’Acqua et al. 2015). First, we analyzed normalized WGS read counts within the confidence interval of this QTL as a proxy for CNV, yet the results did not point to any CNV differentiating the F7 haplotype from the other founders (Supplementary Table S9). Using the same WGS data, we developed consensus sequences for each founder haplotype at this locus to identify putative nonsynonymous substitutions in the protein-coding region, as well as any other sequence variants present in 1 kb downstream and upstream of the relative position of the gene model. The observed absence of non-synonymous substitutions supports the hypothesis that poor *CP24* expression in F7 is not caused by direct alterations to the protein-coding sequence of the gene. Four *cis*-genic allelic variants exclusive to the F7 haplotype were identified (Fig. 6F, Supplementary Table S10). One variant was found downstream of *CP24*, 1 was positioned upstream, and 2 (a nucleotide substitution and a 3 bp deletion) were located within the 5′ and 3′ UTR respectively. Potential transcription factor binding sites (TFBS) were identified using the MEME suite and annotated in the consensus sequence of the *CP24* allele in each of the founder lines. Three of the unique F7 variants were observed to overlap with regions that affect predicted TFBS. The variant located upstream of the gene model, resides in a region with accessible chromatin based on ATAC-seq data from Ricci et al. (2019), and introduces a binding site for *ABI4* which is unique to the F7 haplotype (Fig. 6F; Supplementary Table S11). Furthermore, the variants in the 5′ and 3′ UTRs also impact the predicted TFBS pattern in F7, while the deletion downstream of the gene model does not result in a unique TFBS status in F7 (Supplementary Table S11).

## Discussion

Natural genetic variation in photosynthetic traits provides the starting point for non-transgenic strategies aiming to achieve improved photosynthetic efficiency in crops (Lawson et al. 2012). Here we used a high-throughput protocol to characterize induction and relaxation of NPQ and ΦPSII across 311 maize MAGIC RILs and founder lines grown in a maritime climate close to the northern latitude edge of maize cultivation in the UK (52.2 °N, 0.1 °E), where maize acreage has expanded considerably in recent decades. Understanding the genetics underpinning limitations to photosynthetic efficiency under such conditions is important as maize cultivation expands further into northern Europe. The results from our study confirm the existence of large heritable variation across a range of measured and calculated NPQ and PSII efficiency traits. Below we discuss the implications of our findings in the context of the genetic architecture of these traits, and the potential utility of the identified allelic variation for improving photosynthetic efficiency in maize.

### Variation of ΦPSII and NPQ in the MAGIC maize population is predominantly determined by allelic variation at 4 loci

The kinetics of NPQ induction and relaxation can impact the performance of photosynthesis under fluctuating light conditions, an important determinant of photosynthetic efficiency under field conditions (Kaiser et al. 2018; Long et al. 2022). Natural genetic variation for induction and relaxation kinetics of NPQ and ΦPSII holds promise for crop improvement strategies (Murchie et al. 2018), yet only a few studies have assessed these traits in large field-grown populations to identify genetic associations. Wang et al. (2017) measured NPQ levels on a diverse collection of field-grown rice accessions leading to GWAS-based identification of 33 unique associated SNPs, some of which were validated using bespoke F2 populations.

With respect to C4 species, recent studies have evaluated variation in the kinetics of NPQ and ΦPSII across a large diversity panel of 804 unique maize accessions (Sahay et al. 2023) and across 2 independent sorghum diversity sets, 1 incorporating 374 accessions and the other incorporating 911 accessions (Sahay et al. 2024). The associated field trials were carried out in the traditional maize-growing region of the US Midwest (Lincoln, NE). The broad-sense heritability and the range of variation of measured traits were similar between Sahay et al. (2023) and our study, yet we mapped a smaller number of loci associated with the determination of ΦPSII and NPQ, which is likely a reflection of the reduced genetic diversity captured by the MAGIC population (Scott et al. 2020). The study of Sahay et al. (2023) yielded 18 unique SNPs significantly associated with at least 1 trait and an additional 185 unique SNPs associated at a lower significance threshold, suggesting the existence of a multitude of small-effect genetic loci. Within the confidence intervals of our QTL there were 3,064 protein-coding genes, whereas Sahay et al. (2023) identified a total of 229 protein-coding genes through their GWAS approach. In total, 20 genes were common between the 2 studies (Supplementary Tables S12 and S13). The Arabidopsis orthologs of these genes have not previously been demonstrated to have roles in photosynthesis. These similarities and discrepancies may reflect the difference in environment, genetic materials considered as well as fundamental characteristics of our genetic mapping approach. The MAGIC design used here allows high mapping power with a relatively low number of distinct genotypes, alleviating the phenotyping bottleneck for traits that are difficult to measure in sufficiently high throughput (Dell’Acqua et al. 2015). The MAGIC design also comes with the advantages of low population structure and fully resolved identity by descent (Scott et al. 2020), which enable detailed further study of haplotype effects on trait determination and candidate genes mapping (Scott et al. 2020). For the results presented here, the mapped QTL contain tens to hundreds of gene models (Tables 1 and 2), hence it becomes paramount to prioritize candidate genes within the mapping intervals.

### Identification of candidate genes in QTL mapping intervals

One potential means for genetic variants to give rise to QTL effects is via impacting transcription of genes via variation in proximal or distal regulatory elements. Transcriptome-wide association study (Gamazon et al. 2015) has been developed to identify transcriptionally regulated genes in association with complex traits, which in combination with GWAS or QTL mapping can help prioritize causal genes by means of colocalization, as demonstrated recently for water use efficiency trait variation in *Sorghum bicolor* (Ferguson et al. 2021). Here, we used preexisting expression data for the founder lines specifically for the gene models contained within each QTL interval to test for correlations between gene expression level variation and QTL effects by founder haplotype. This strategy filters for genes which are consistent with the specific case where a genetic variant associates with both the expression level of a causal gene and the trait of interest. It should be, however, emphasized that this is only 1 nonexclusive mode of action to explain QTL effects, as variation in a trait may not be due to the difference in expression levels and expression levels at the fourth leaf stage, as used in this study, may not be maintained in the adult plant in which the phenotypic measurements were conducted. Nevertheless, 10 genes were found to be potentially in agreement with this case, i.e. for which previously determined expression levels were significantly correlated with clustered QTL effects. Of these 10 genes, the presence of phenotypes in knockout mutants of the orthologous genes in Arabidopsis consistent with the corresponding QTL effects can be interpreted as evidence in favor of a causative role of an uncharacterized phosphofructokinase and a protein capable of reversible glycosylation (*RGP2*) in regulating NPQ, but not as conclusive proof. The mechanisms underlying this regulation are also not immediately clear from the annotated functions of these genes or gene families, which therefore provides interesting targets for further study. A further means of candidate gene prioritization was provided through the local GWAS approach which enabled the identification of 2 significant SNP-trait associations where the SNPs lay within a gene model (Fig. 5, Supplementary Table S6). Through this approach we identified a chloroplastic protein kinase where the phenotypes of Arabidopsis mutants for the 3 genes with high sequence similarity to the identified maize gene were consistent with a role in NPQ induction (Supplementary Fig. S10). Previous work has highlighted a role for members of this gene family in stomatal responses to light (Elhaddad et al. 2014). The tentative identification of this maize chloroplastic protein kinase as a candidate gene here may be reflective of proposed associations between guard cell electron transport and chloroplast redox regulation with stomatal behavior (Lemonnier and Lawson 2024).

The shared large-effect QTL for ΦPSII and NPQ on chromosome 10 was primarily driven by the negative founder effect of the F7 founder haplotype (Fig. 4, G and H). This effect was validated by further measurements on the F7 founder line, which showed remarkably low *CP24* expression levels and protein accumulation, decreased supercomplex abundance, and fast fluorescence rise time (Fig. 6). *A. thaliana* T-DNA insertion mutants of the additional 4 genes that showed expression patterns matching QTL founder effects did not have any effect on dark-adapted maximal PSII efficiency estimated by *F*_v_/*F*_m_ (Supplementary Fig. S9), thereby further increasing confidence in *CP24* as the causal gene. *CP24* (*LHCB6*) is a monomeric light-harvesting complex protein which is positioned between the moderately bound LHCII trimers and the core complex. Nevertheless, excitation energy transfer from *CP24* is predicted to occur via the moderately bound LHCII to the core PSII complex (Croce and Van Amerongen 2020). *CP24* seems to have evolved in land plants and has been lost in some subgroups of gymnosperms (Koziol et al. 2007; Kouřil et al. 2016). Previous work on *Arabidopsis thaliana* knock-out mutants of *CP24* shows a pronounced reduction of *F_v_/F_m_*, NPQ amplitude and antenna supercomplex abundance, which also manifests in reduced growth rates (Kovács et al. 2006; de Bianchi et al. 2008). Additionally, it was observed that *CP24* deficiency may result in PSII particles becoming organized into tightly packed arrays which may limit protein dynamics and plastoquinone diffusion (de Bianchi et al. 2008). This may therefore form the mechanistic basis for the Chr 10 QTL effect of the F7 allele observed here. Significant *CP24* downregulation has been observed in Arabidopsis in response to high light (Ballotari et al. 2007; Floris et al. 2013) which may suggest that F7-specific post-transcriptional regulation could have contributed to the observed *CP24* deficiency. However, the strong decrease in *CP24* transcript levels in F7 seems to suggest that the deficiency does not primarily result from translational regulation. Whilst we found preliminary evidence that allelic variation at TFBS may be responsible, the specific genetic mechanism of the *CP24* deficiency in F7 remains unresolved.

### Potential utility of identified QTL for maize improvement

*F*_v_/*F*_m_ and ΦPSII are robust indicators of the maximum quantum yield and the operational efficiency of PSII photochemistry respectively (Baker 2008; Murchie and Lawson 2013). ΦPSII is tightly linked to the quantum yield of CO_2_ fixation (ΦCO2) measured by gas exchange in maize (Fryer et al. 1998; Pietrini and Massacci 1998; Leipner et al. 1999) and other C4 species (Cousins et al. 2002; Ripley et al. 2007) across a range of environmental conditions. This suggests that the QTL for ΦPSII on chromosomes 9 and 10 are quite likely to impact the efficiency of photosynthetic CO_2_ assimilation, which could consequently have implications for yield (Wu et al. 2019). F7, which harbors the negative ΦPSII chromosome 10 allele, is an important founder line for European breeding programs (Unterseer et al. 2017) (Supplementary Fig. S12 and Table S14). F7 is an early-flowering flint line and has historically been used to breed for earliness, which has been actively selected for in temperate regions to compensate for shorter growing seasons (Soengas et al. 2006). Interestingly, Mayer et al. (2020) recently discovered a haplotype that co-localizes with our negative chromosome 10 QTL as part of a characterization of double haploid lines derived from 3 European flint maize landraces. This haplotype has substantial and unfavorable effects on vigor and plant height at multiple developmental stages and across 11 independent environments, which may be aggravated by reduced photosynthetic rates.

Although NPQ is less directly related to CO_2_ assimilation, it has been shown to transiently limit ΦCO_2_ in C3 species like *Nicotiana tabacum* (Kromdijk et al. 2016) and *Glycine max* (De Souza et al. 2022). Several recent studies have demonstrated variation in NPQ induction and relaxation in C3 species (Rungrat et al. 2019; Cowling et al. 2022; Theeuwen et al. 2022). Direct comparisons of NPQ kinetics between these studies and ours are complicated by the use of different phenotyping protocols and the use of different methods for modeling the response of NPQ to illumination and darkness. Despite this, it seems that NPQ is generally slower to relax in the aforementioned C_3_ studies compared with NPQ in the C_4_ crop maize in this study and in Sahay et al. (2023). Although substantial variation remains, this may suggest that NPQ kinetics are generally already closer to optimal in maize. Nevertheless, manipulation of photoprotection via enhanced NPQ amplitude may also have benefits for yield (Hubbart et al. 2018) and intrinsic water use efficiency (Głowacka et al. 2018; Turc et al. 2024) independent from NPQ kinetics.

For the identification of QTL or SNPs in collections of highly homozygous accessions in diversity panels or mapping populations to be relevant for improvement of photosynthesis in new maize cultivars, the associations need to remain important in heterozygous backgrounds. Maize is a model species for heterosis (Shull 1948). Indeed, plant breeding vastly enhanced maize yield by exploiting the positive effects of heterosis on plant size and growth rate. However, despite the importance of heterosis, its molecular basis and underlying genetic mechanisms are still largely unclear, notwithstanding some recent progress (Xiao et al. 2021). The level of heterosis varies strongly between traits and determines the ability to predict hybrid performance based on the inbred founder phenotypes, where traits with greater heterosis show relatively weaker correlations (Flint-Garcia et al. 2009). Evidence for heterosis effects on photosynthetic traits is inconsistent. Recent work in *A. thaliana* found heterosis effects on photosynthetic traits to be negligible across 4 sets of reciprocal F1 hybrids from contrasting ecotypes, despite strong heterosis effects on biomass accumulation (Liu et al. 2020). In maize, some work indicates that heterosis may impact photosynthetic traits (Mehta and Sarkar 1992; Meena et al. 2021), whereas others report a much lesser impact (Kamphorst et al. 2022). Maize homozygous lines suffer from inbreeding depression due to the accumulation of deleterious mutations, and cultivated maize germplasm segregates for many SNPs predicted to result in deleterious variants (Mezmouk and Ross-Ibarra 2014), possibly including negative alleles for photosynthetic efficiency such as that in F7. While the heterotic potential of the identified QTL remains unclear, it is possible that these alleles still have an effect in a heterozygous background. Future research will therefore need to include validation of the QTL effects in a heterozygous state.

## Conclusions

Genetic variation in leaf photosynthetic traits may have promise to enhance photosynthetic efficiency in maize. Here, genetic determinants of variation in photosynthesis and NPQ were mapped by phenotyping a collection of maize MAGIC RILs under temperate field conditions. Most trait variation was captured by 4 major QTL, for which potential candidate genes were identified using a range of approaches, which identified 4 high priority candidate genes for further testing. *CP24* was identified as the highest confidence candidate gene for a large effect QTL for photosynthetic efficiency and NPQ on the distal end of chromosome 10, driven by poor *CP24* expression associated with the haplotype of the F7 founder line. The historical role of this line in breeding for early flowering time may suggest that the presence of this poorly expressing allele could be enriched in temperate maize germplasm.

## Materials and methods

### Plant material and field experiments

The MAGIC maize population described in Dell’Acqua et al. (2015) was used in this study. This population was developed by crossing 8 inbred lines (A632, B73, B96, F7, H99, HP301, Mo17, and W153R) and following a funnel breeding design. A ninth line (CML91) was introduced into the design as a 2-way hybrid (B73xCML91) to account for failures in crossing B96xHP301. For this study, we used a subset of 320 RILs, different from those in the original paper, that well-represent the genetic composition of the population (Supplementary Fig. S13).

Field experiments were carried out in 2021 and 2022 at the National Institute of Agricultural Botany (NIAB, Cambridge, UK) on heavy clay loam soil types. The field experiment sites were approximately 500 m from each other across the 2 yr (Supplementary Fig. S1). Environmental data (precipitation, temperature, and light intensity) were collected from the same weather stations across the 2 yr (Supplementary Fig. S2).

In each year, 320 RILs were grown in an alpha-lattice experimental design with 2 replicates of 40 blocks each, where each block contained 8 plots. The experimental design was constructed using the design.alpha function of the R/agricolae package (Mendiburu and Simon 2015). Each plot consisted of 4 rows of 10 plants representing a single RIL. The rows were spaced 25 cm apart and each plant within a row was 13 cm apart. In both years, seeds were sown by hand at a depth of 10 to 15 cm. 301 RILs were common between each year (Supplementary Table S1).

The 2021 experiment was sown on May 13, 2021 and the 2022 experiment was sown on 04/05/2022. Irrigation inputs were provided for the 2022 experiment only (Supplementary Fig. S2). In both years, pesticides and fertilizers were applied throughout the experiment as necessary and according to manufacturer instructions (Supplementary Table S15).

### Chlorophyll fluorescence phenotyping

Six plants per RIL were measured, with 3 random plants coming from the representative plot in the first replicate of the alpha-lattice experimental design and 3 random plants coming from the second replicate. Measurements of chlorophyll fluorescence were performed on the leaf subtending the ear between 3 and 8 d postsilking. Our phenotyping focused on the leaf subtending the ear, since photosynthesis of this leaf is particularly associated with grain yield following flowering (Cagnola et al. 2021). Further, we tried to phenotype all leaves within a 3-to-8-d window following silking to minimize any unwanted variance due to the onset of leaf senescence (Wedow et al. 2021). Any variance that did occur within this window was statistically accounted for as described later.

Measurements were spread out across 3 wk in each year to account for the variation in silking time.

On each measurement day, the leaf subtending the ear from 3 plants from all plots selected to be measured that day was excised at the base. The excision point was then immediately submerged in water. This was performed at dawn (∼0530 h) within a 30-min window. The leaves were then returned to the laboratory and recut under water to maintain the water column and left in a 50 mL Falcon tube under stable conditions for 7 h. After this time, a 2 cm × 4 cm strip of tissue was cut from the middle of the leaf and placed on top of damp filter paper that was on top of a non-reflective glass plate as per (Ferguson et al. 2020). 60 to 70 cut tissue samples were placed together on 1 sheet of paper in a grid-like fashion. A reference map of each grid of samples was made using QR codes attached to each leaf to cross-reference the data with the sample it was generated from. Once all samples had been laid out, a non-reflective glass plate was placed on top of the samples to keep them flat and in place and to ensure that they did not desiccate. Samples were then dark-adapted overnight using aluminum foil before performing chlorophyll fluorescence measurements in the following morning at 0900 h. This experimental routine allowed us to perform additional measurements on the same leaf material prior to excising the tissue for chlorophyll fluorescence. We have recently demonstrated that the data generated for NPQ kinetics and maximum PSII operating efficiency through this approach is identical to that produced by directly measuring leaves that are still attached to the plant in maize (Ferguson et al. 2023).

Chlorophyll fluorescence measurements were performed using a closed chlorophyll fluorescence imaging system (FluorCam FC 800-C, PSI, Czech Republic). Initially, the measuring beam was switched on to estimate minimal chlorophyll fluorescence (*F*_o_). A saturating light pulse was then used to calculate dark-adapted maximum fluorescence (*F*_m_). The actinic light source (1500 µmol m^−2^ s^−1^ photosynthetic active radiation [PAR]) was then switched on for 10 min. During these 10 min, a series of 12 saturating pulses were performed to estimate maximal fluorescence (*F*_m_′) at the following intervals (in seconds): 20, 40, 60, 120, 180, 240, 300, 360, 420, 480, 540, 600. The light source was then subsequently switched off for 12 min and a further series of 8 saturating pulses were performed to again estimate *F*_m_′ at the following intervals: 20, 40, 60, 120, 180, 360, 540.

Using the above-described data, the maximum quantum efficiency of PSII photochemistry was calculated as *F*_v_/*F*_m_ where *F*_v_ is the variable fluorescence between *F_m_* and *F*_o_. At each point of measurement following the initial dark-adapted saturating pulse, NPQ was calculated as (*F*_m_-*F*_m_′)/*F*_m_′ and PSII operating efficiency (ΦPSII) was calculated as (*F*_m_′-*F*′)/*F*_m_′ (Fig. 1; Murchie and Lawson 2013). If the *F*_v_/*F*_m_ value for any sample was below 0.70, it was removed from the analyses. This was done to ensure that samples were not included where something other than underlying genetics was limiting photosynthesis and resulted in less than 2% of all samples being removed.

The slope from a linear model describing NPQ as a function of time was used to characterize the initial (0 to 80 s) response of NPQ to actinic light (NPQ induction slope; Fig. 1B). Exponential models were used to characterize the induction of NPQ in response to the actinic light being switched on (Equation 1; Fig. 1C), the relaxation of NPQ following the actinic light being switched off (Equation 2; Fig. 1D), and the recovery of ΦPSII following the turning of the actinic light (Equation 3; Fig. 1F).


(1) $$\mathrm{NPQ}=a\;\times (1-\mathrm{ex}p^{\left(-b\times t\right)})$$

Where *a* represents the amplitude (NPQ induction amplitude), *b* represents the rate constant for the induction of NPQ (NPQ induction rate), and t represents time in seconds.


(2) $$\mathrm{NPQ}=a\;\times (\exp^{\left(-b\times t\right)})+c$$

Where *a* represents the amplitude (NPQ relaxation amplitude) and *b* represents the rate constant for the induction of NPQ (NPQ relaxation amplitude). Here, *c* represents an offset to account for NPQ not reaching zero during this relaxation phase (NPQ relaxation offset).


(3) $$\Phi \mathrm{PSII}=a\;\times \;(1-\exp^{\left(-b\times t\right)})+c.$$

Where *a* represents the amplitude (ΦPSII recovery amplitude) and *b* represents the rate constant for the recovery of ΦPSII (ΦPSII recovery rate). Here, *c* represents an offset to account for a non-zero intercept (ΦPSII recovery offset).

We also extracted the maximum value for NPQ as well as the final values for NPQ and ΦPSII.

Finally, we also calculated thePI (Equation 4) which is a modification of the “pNPQ” approach of (Ruban and Murchie 2012)


(4) $$\mathrm{PI}=\;\frac{\Phi \mathrm{PSIIf}}{1-\left[\frac{\left(1-\frac{F_{v}}{F_{m}}\right)}{\left(\frac{F_{v}}{F_{m}}+\left[\frac{\left(1-\frac{F_{v}}{F_{m}}\right)}{\frac{1}{1+\mathrm{NPQf}}}\right]\right)}\right]/\frac{1}{1+\mathrm{NPQf}}}$$

For Equation (4), *Φ*PSIIf and NPQf are the final dark time point values for ΦPSII and NPQ, respectively.

### Statistical analyses

We extracted best linear unbiased predictors (BLUPs) for all measured traits from a general linear mixed model (Equation 5) performed via restricted maximum likelihood using the lmer and ranef functions from the lme4 R package (Bates et al. 2015) with the following formula with effects treated as random:


(5) $$\begin{array}{l} \mathrm{phenotype}=\mathrm{overall}\;\mathrm{mean}+\mathrm{genotype}+\mathrm{year}\;+\\ \;\mathrm{year}:\mathrm{genotype}+\mathrm{year}:\mathrm{rep}+\mathrm{year}:\mathrm{rep}:\mathrm{block}+\mathrm{year}:\mathrm{date}\;+\\ \;\mathrm{year}:\mathrm{silking}\;\mathrm{interval}\;+\mathrm{error}\;\mathrm{variance} \end{array}$$

BLUP values were added to the population mean to generate predicted means for each trait. To extract year specific BLUPs, models identical to Equation (5) were performed but without the year terms and including only data from either 2021 or 2022. Broad sense heritability ($H_{B}^{2}$) for each trait was calculated from the coefficients from the corresponding models as the ratio of genotypic variance to phenotypic variance according to the standard method (Schmidt et al. 2019).

Correlations between identical traits across 2021 and 2022 were tested for statistical significance via Pearson correlation analyses using the cor.test function in R. Correlations between all possible pairwise trait interactions on a year-by-year basis were also tested in this manner. One-way analysis of variance (ANOVA) comparison of means tests was performed to test for significant differences in the variance of each measured trait across 2021 and 2022 using the aov function in R.

### DNA extraction, genotyping, and bioinformatic analysis

Genomic DNA of founders and RILs was extracted by bulking young leaves of 3 seedling per genotype with the GenElute Plant Genomic DNA Miniprep Kit (Sigma Aldrich, Germany) following the manufacturer's protocol. Integrity was assessed with agarose gel electrophoresis; DNA samples were quantified with the Qubit fluorometer (Invitrogen, Thermo Fisher Scientific, US). Samples were delivered to IGA Technology Services (Udine, Italy) to perform genotyping with the Single Primer Enrichment Technology (SPET), a protocol using single primer extension reactions to enrich pre-defined target loci. The set of SPET probes was developed starting from MAGIC maize founder haplotypes to maximize evenness of representation (Scaglione et al. 2019). Libraries were prepared with the Illumina TruSeq DNA Protocol (Illumina Inc., San Diego, CA, United States) and targeted fragments were sequenced with V4 chemistry in paired-end 150-bp mode on an Illumina HiSeq 2500 (Illumina Inc., San Diego, CA, United States). After initial quality assessment (FastQC), raw reads were filtered with erne-tool (ERNE2 package, version 2.1.1, http://erne.sourceforge.net/; (Del Fabbro et al. 2013). Trimmed reads were then mapped against the *Z. mays* reference genome (RefGen V4, Jiao et al. (2017)) with the BWA-MEM algorithm (Li and Durbin 2009). The HaplotypeCaller algorithm (GATK) was used for variant call (Poplin et al. 2018). Genotyping was completed with GenotypeGVCFstool (Danecek et al. 2011) to derive raw single nucleotide polymorphisms (SNPs). SNPs were filtered using a Phred-scaled score greater than 30 and a SNP missingness per individual and locus lower than 20%.

### RNA sequencing data

Raw transcriptome data of 8 founder lines except B96 were retrieved from ArrayExpress (https://www.ebi.ac.uk/arrayexpress/) under accession number E-MTAB-3173. Briefly, these data were generated on the MAGIC founder lines grown under controlled growth conditions: 24 °C, 55% relative humidity, 170 μmol m^−2^ s^−1^ photosynthetically active radiation at plant level in a 16 h/8 h d/night cycle. Tissue for RNA extraction was sampled at the fourth leaf from 3 biological replicates, during the steady-state growth phase. Samples were extracted with Trizol (Invitrogen), library prepared, and sequenced with 3 technical replicates as detailed by Baute et al. (2016). Raw reads were subjected to QC with ERNE-filter tool (ERNE2 package) and mapped against RefGen V4.0 (Jiao et al. 2017) using STAR v. 2.5.3a (Dobin et al. 2013) in 2-pass protocol, with default parameters (Miculan et al. 2021). Transcript abundances were extracted and normalized using R/edgeR (Robinson et al. 2010). For QTL where founder effects were distinct between B73 and F7, we additionally queried gene expression difference between these 2 founders utilizing the RNAseq datasets described in Cackett et al. (2023).

### Mapping of QTL and candidate gene identification

The genetic map was derived by anchoring the intermated B73 x Mo17 (IBM) genetic position over the SNPs derived after QC on the RefGen V4. Missing genetic distances were interpolated using values proportional to the physical distance. The reconstruction of the founder’ haplotypes in the RILs genomes (i.e. estimation of genotype probabilities) was executed using a Hidden Markov Model (HMM), implemented in R/qtl2 (Broman et al. 2019) that was used as platform for all the following mapping steps when not stated otherwise. QTL mapping of the phenotypes was performed using a mixed linear model incorporating kinship through the “Leave One Chromosome Out” (LOCO) method. The genome scan regressions logarithm of odds (LOD) scores were estimated using the sum of squared residuals for the null and alternative hypotheses. Thresholds of significance were defined based on 999 trait-specific permutations. The 95^th^ percentile of the permuted LOD distributions was set as threshold to identify peaks. For each of the significant LOD peaks, we used Bayesian credible interval estimation to define QTL confidence intervals, which were expanded to the closest SNP marker physical positions.

For each peak, we estimated QTL coefficients within the confidence intervals, which represent founder haplotype effects as estimated by the model. The model conducts the mapping using founder haplotypes reconstructed on RIL genomes via HMM, and the haplotype effects estimates derive from phenotypic values observed in RILs carrying a given founder haplotype in the locus of interest. QTL coefficients in the confidence interval were clustered using a k-means algorithm with a function implemented in R/fpc (Hennig 2010), that searched for the optimal number of clusters ranging from one to the number of founders except one. This procedure enabled us to identify those founders or group of founders that showed contrasting effects at each QTL, i.e. those founders that was contributed to positive or negative trait values. We then used this information to prioritize gene models within the QTL mapping intervals based on gene expression levels measured on founder lines at the fourth leaf stage, under the assumption that constitutive differences in gene expression levels in founders are transferred to RILs and contribute in the determination of the trait. For each QTL, we used the most divergent groups of founder effects (clusters) as a factor level to test for differential expression of genes within each corresponding confidence interval. In other words, we checked whether those founders most contributing to trait levels had significantly high (or low) expression level of any of the genes in the QTL mapping interval. We tested these differences, for each gene, with a generalized linear model fitting transcript count data derived from RNA sequencing on founder lines with the founder effect groups. We then used a threshold of FDR 0.1 was used for multiple testing correction arising from the individual gene tests. This approach has the advantage of rapidly detecting suggestive candidate genes within QTL mapping intervals even when hundreds are present. It is based on the following assumptions that are important to specify: (i) transcript levels contribute to the expression of the trait; (ii) differences in transcript levels across founder lines are constitutive and can be detected at the fourth leaf stage; (iii) transcript levels of founder lines are maintained in RILs. This method will only identify gene models that match all these assumptions. Arguably, QTL may be contributed by other mechanisms that this method is not able to capture. Due to the low number of RILs carrying the CML91 haplotype at the QTL, their effect as well as transcript abundances were excluded from these analyses (Dell’Acqua et al. 2015). The search for differentially expressed genes, based on founders' haplotype effects, was only applied to significant peaks having confidence intervals with a span of less than 30 Mbp. Once we identified candidate genes with this method, we searched the most similar Arabidopsis ortholog based on putative protein sequence identity. The procedure was carried out using the freely available database in PhytozomeV13 (Goodstein et al. 2012) as well as Plaza Monocot v. 4.5 (Van Bel et al. 2018).

To further the characterization of the identified QTL, we employed a R/qtl2 built-in procedure designed to test marker associations within specific regions. This approach collapses genotype probabilities of the RILs to the SNPs of the founder genotypes at a single QTL region. For this analysis, we retrieved a comprehensive WGS SNP database which was made available for the founders of our population (Dell’Acqua et al. 2015). From this database we derived 4,474,859 homozygous, polymorphic SNP. With this resource, we imputed founder SNPs onto the reconstructed RIL genomes. This way we were able to test associations in a given QTL, using a standard mixed linear model regression that incorporates kinship with the aforementioned LOCO method. To ensure adequate statistical power and a sufficient number of SNP markers within each identified QTL, the confidence intervals were extended by 250 kbp both upstream and downstream. The most significant marker within these extended intervals was used to prioritize candidate genes, but only if it met a Bonferroni-corrected threshold with an alpha level of 0.05.

To fully exploit the genetic features of the MAGIC maize population we realigned existing WGS short-read sequencing data produced on the founders of the population (Dell’Acqua et al. 2015) against RefGen V4 using BWA-MEM. Before alignment all reads were quality-controlled by Fastqc v0.12.1 (Andrews 2010) and filtered with bbduk from bbtools v38.87 (Bushnell 2015) to gain reads with a quality of at least 25 and minimum length of 35 bp. Bam files were filtered for a minimal mapping quality of 30 and sorted and indexed with samtools v1.17 (Danecek et al. 2021). Mapping statistics were called with qualimap v.2.2.2-dev (García-Alcalde et al. 2012). CNVs were determined with cn.mops v1.46.0 package (Klambauer et al. 2012) based on read counts in 100 K bp bins for each chromosome. This approach was used to provide insights into structural variation as inferred CNV within QTLs confidence intervals.

### Investigation of potential determining factors for CP24 deficiency in F7

We narrowed down our attention to the coding region of CP24 including 1 kbps upstream and downstream the relative gene model position. In the first step, our aim was to identify non-synonymous substitutions. To the purpose, we annotated the variants of all the founders with SnpEff v5.2-0 (Cingolani et al. 2012). Secondly, TFBS profiles of the founders in this region were annotated to identify unique patterns characterizing the F7 haplotype. To the purpose, consensus sequences of the founders were obtained with samtools faidx v0.1.19 and bcftools consensus v1.14 (Danecek et al. 2021). The consensus sequences were then aligned in a multiple sequence alignment (MSA) with mafft v7.520 (Katoh and Standley 2013). Potential TFBS were determined for each founder consensus sequence through ‘Find Individual Motif Occurrences' FIMO v5.5.4 (Grant et al. 2011) using the jaspar_core_plant_single_batch_nr_db.meme database (Sandelin et al. 2004). To identify regions of accessible chromatin, we retrieved ATAC-seq data in the proximity of the coding region of CP24 from data produced by Ricci et al. (2019).

### Phylogenetic tree reconstruction for ZmKEA1, −2, −3

Phylogenetic reconstruction of the ZmKEA1, −2, −3 was carried out using the online platform PLAZA monocot (version 5) (Van Bel et al. 2021). Sequences for ZmKEA1, ZmKEA2, and ZmKEA3 were obtained through MSA of the protein sequence *Zm00001d026645* against the B73 maize reference genome as well as the Arabidopsis reference available on PLAZA monocot (version 5). The MSA was conducted with the MUSCLE software (version 3.8). The phylogenetic tree was then constructed with FastTree (version 2.1), with the *-wag* and *-gamma* options.

### Pedigree analyses

Pedigree analyses were carried out using the MaizeGDB Pedigree Viewer. The tool is based on a pedigree network of 4,706 maize varieties that are currently available in the MaizeGDB Stock Pages.

### Additional chlorophyll fluorescence phenotyping of B73 and F7 founders

Characterization of chlorophyll fluorescence parameters related to PSII antenna size and efficiency was performed using the LCF fluorometer of LI-6800 Portable Photosynthesis System (LI-COR Biosciences, USA). OJIP induction kinetics elicited by a 600 ms square pulse of 20,000 µmol m^−2^ s^−1^ irradiance were monitored using the continuous fluorescence signal mode in order to achieve the best signal-to-noise and temporal response (∼1.6 MHz bandwidth detection response with 4 μs). The relative change of fluorescence yield was derived from the ratio of the continuous fluorescence signal divided by the continuous actinic irradiance signal monitored by the photodiode used for pulse optical control (Loriaux et al. 2013). The boosted flash intensity promoted fluorescence rise from *F*_o_ level to maximal *F*_j_ level and the corresponding reduction of the primary PSII quinone acceptor (Q_A_) within the first few turnovers of PSII. The half rise time *t*_1⁄2_ defined as the time to rising from *F*_o_ to one-half of (*F*_j_-*F*_o_) was used as a proxy of the rate of photochemical reduction of Q_A_ and the reciprocal of *t*_1⁄2_ provided a relative estimate of the effective antenna size of PSII. The measurement was carried out on single leaf that was dark adapted for at least 30 min.

### Phenotyping of Arabidopsis T-DNA insertion mutants

All mutant lines were generated from *Arabidopsis thaliana* wild-type Columbia-0 (Col-0) or Columbia-3 (Col-3). *Arabidopsis* mutant lines for the following genes were obtained from the Nottingham Arabidopsis stock center: *APK1A* (SALK_056259C), *AT1G76550* (SAIL_1151_H05), *AT2G17410* (SALK_026835C), *AT2G20760* (SALK_133492C), *AT4G27450* (SALK_046196C), *AT5G02430* (SALK_136212C), *CDC48* (SALK_005957C), *CDL1* (SALK_039503C), *KEA1* (SAIL_1156H07), *KEA2* (SALK_045324), *PK1B* (SALK_001115C), *RGP2* (SALK_132152C) and *VTE4* (SALK_036736C). All mutants were validated as homozygous via PCR following standard practice (O’Malley et al. 2015). Primers used are listed in Supplementary Table S16. All plants were initially grown for 6 wk under short day conditions (8 h light/16 h dark, 22 °C, 60% humidity, 150 µmol m^−2^ s^−1^) and then shifted to a growth cabinet with a 12-h light/dark cycle and similar conditions at least 2 d before experiments. Whole rosettes were phenotyped via chlorophyll fluorescence exactly as previously described for the maize leaf sections. The plants were placed on a dimly lit lab bench for >1 h and then fully dark adapted for 15 min before initiation of the chlorophyll fluorescence phenotyping. Between 8 to 15 biological repeats were measured per unique mutant and wild type. All plants where data are presented together were grown in the same batch. Statistical differences between mutant lines and wild type (either Col-0 or Col-3) were determined via *t*-test (alpha = 0.05). Instances where *P*-values were less than 0.05 and thereby indicating a statistically significant difference are highlighted using red asterisks. For all Arabidopsis data, we provide the means (and standard errors) for initial *F*_o_ and *F*_m_ (Supplementary Figs. S6 and S7 and S9, and S10). In all cases, there were no significant differences in either *F*_o_ or *F*_m_ between mutants and associated wild types. A summary of all *t*-test results is provided in Supplementary Table S17.

### Protein analyses

Thylakoid membrane protein complexes were isolated according to (Järvi et al. 2011). Briefly, 1 g of frozen leaf material (fourth fully expanded leaf) was ground in ice-cold grinding buffer (50 mm HEPES/KOH, pH 7.5, 330 mm sorbitol, 2 mm EDTA, 1 mm MgCl_2_, 5 mm sodium ascorbate, 0.05% BSA, 5 mm NaCl, and 10 mm NaF) and filtered through 2 layers of Miracloth (Sigma Aldrich). The flow-through was spun down for 5 min at 5000 × g at 4 °C, and the resulting pellet was resuspended in 2 mL shock buffer (50 mm HEPES/KOH, pH 7.5, 5 mm sorbitol, 5 mm MgCl_2_, and 10 mm NaF) and then in 2 mL storage buffer (50 mm HEPES/KOH, pH 7.5, 100 mm sorbitol, 10 mm MgCl_2_, and 10 mm NaF). Finally, the pellet was carefully resuspended in 100 µL storage buffer. All the steps were performed under dim light and on ice. The chlorophyll concentration was determined from a 5 µL sample in 100% methanol according to (Porra et al. 1989). Aliquots of the thylakoid fractions were diluted with ACA buffer (50 mm BisTris/HCl (pH 7.0), 750 mm ε-aminocaproic acid, 1 mm EDTA, 0.25 mg ml^−1^Pefabloc, and 10 mm NaF) to 1 µg chl µL^−1^ and stored at −70 °C.

For western blot analysis, 2 µg chl of thylakoid samples were denatured for 5 min at 70 °C in 2× Laemmli sample buffer (138 mm Tris/HCl (pH 6.8), 6 m urea, 22.2% (v/v) glycerol, 4.3% (w/v) SDS, 5% (v/v) 2-mercaptoethanol, and 0.05% bromophenol blue). Samples were briefly spun down (5000 × *g* for 5 min), loaded onto a 12% Mini-PROTEAN TGX Precast Protein Gel, and run in a Mini-PROTEAN Electrophoresis Cell (Bio-Rad) using a Tris/glycine running buffer (25 mm Tris base, 190 mm glycine, and 0.1% SDS). Proteins were then blotted onto a PVDF membrane (pore size 0.2 µm) using a Trans-Blot Turbo Transfer System (Bio-Rad) and the “Mixed MW” protocol (7 min blotting time). The membrane was briefly washed in TBS buffer and then blocked with 10% nonfat milk in T-TBS buffer for 1 h on a shaker. After that, the membrane was washed 2 times for 5 min with T-TBS on a shaker and incubated in the primary antibody (anti-CP24 (AS01 010), Agrisera, 1:2,500 in 1% milk in T-TBS) overnight at 4 °C, shaking. The next day, the membrane was washed 4 times for 5 min with T-TBS and incubated in the secondary antibody (AS09 602, Agrisera, goat anti-rabbit IgG, HRP conjugated, dilution = 1:12,500 in 1% milk in T-TBS) for 1 h on a shaker. Finally, the membrane was washed 3 times with T-TBS and twice with TBS for 5 min each and incubated with Clarity Western ECL substrate (Bio-Rad). After 5 min, the blot was imaged using a G:Box Chemi XRQ system (Syngene) with an exposure time of 3.5 min.

For blue native-polyacrylamide gel electrophoresis (BN-PAGE), 10 µg chl samples were solubilized with an equal volume of 2% digitonin (in ACA buffer, final concentration = 1%) by gently shaking samples at room temperature for 10 min. Afterwards, samples were spun down for 20 min at 18,000 × g at 4 °C and the supernatant was transferred into new tubes containing 1/10 of the volume of BN-PAGE sample buffer (100 mm BisTris/HCl (pH 7.0), 0.5 m ACA, 30% (w/v) sucrose, and 50 mg mL^−1^ Serva Blue G). After careful mixing, samples were loaded onto a 3% to 12% Native PAGE Bis/Tris precast gel (Invitrogen) and were run using the XCell SureLock Mini-Cell system (Invitrogen) on ice. The outer chamber was filled with clear anode buffer (50 mm BisTris/HCl, pH 7.0), whereas the inner chamber contained blue cathode buffer (50 mm Tricine, 15 mm BisTris/HCl (pH 7.0), and 0.01% Serva Blue G). Electrophoresis was performed using the following protocol: 75 V for 30 min, 100 V for 5 min, 125 V for 30 min, 150 V for 1 h and 175 V for 60 to 90 min. After the first 90 min, the blue cathode buffer was replaced with a clear cathode buffer, omitting Serva Blue G. The gel was then imaged using a flatbed scanner.

### Analysis of *CCD1* copy number by RT-qPCR

Real-time quantitative PCR (RT-qPCR) was used to determine the copy number of *CCD1* in each founder. Genes of known single copy, *VP14* (Tan et al. 2017) and *ADH1* (Broothaerts et al. 2008) were used as references. Genomic DNA of founders was extracted from young leaves using the CTAB method. To improve the accuracy of the RT-qPCR, genomic DNA was first digested to completeness with EcoRI. Reactions were then prepared using 200 ng digested DNA, 200 nmole of forward and reverse primer sets for reference genes (ADH1,5′-CGTCGTTTCCCATCTCTTCCT-3′ and 5′-CCACTCCGAGACCCTCAGTC-3′; VP14, 5′-GCTGGCTTGGCTTGTATACTCTG-3′ and CCATCAGTCATATACTGTGAACAAATGT-3′) and CCD1 (5′-GGGAAGAGGGTGATGAAGTTGT-3′ and 5′-TGATATCCATTCACCTTGTCCAAA-3′) and 5 µL of SsoAdvanced Universal SYBR Green Supermix (172-5270; BioRad, Hercules, CA, USA). All samples were analyzed in triplicate using the CFX connect Real-Time PCR Detection System (1855201, BioRad, Singapore). The following RT-qPCR conditions were used: 3 min at 98 °C, 40 times (10 s at 98 °C, 30 s at 60 °C), followed by a melting curve from 60 °C to 95 °C. Copy number was estimated using the ΔΔCt method (Livak and Schmittgen 2001) and Ct values were normalized against B73 as a single copy reference (Tan et al. 2017).

### Accession numbers

The raw sequences obtained using SPET, used in this study, are available at the European Nucleotide Archive (ENA) (https://www.ebi.ac.uk/ena/browser/home), under project ID PRJEB67515.

## Supplementary Material

koaf063_Supplementary_Data

## Data Availability

The authors responsible for distribution of materials integral to the findings presented in this article in accordance with the policy described in the Instructions for Authors are Matteo Dell’Acqua (m.dellacqua@santannapisa.it) and Johannes Kromdijk (jk417@cam.ac.uk). The scripts used for data analysis are available on GitHub at https://github.com/capleo/MAGIC.

## Supplementary data

The following materials are available in the online version of this article.

**Supplementary Figure S1.** Comparison of selected parameters across the 2 experimental years.

**Supplementary Figure S2.** Environmental parameters across the 2 experimental growing seasons.

**Supplementary Figure S3.** Broad-sense heritability for the main measured and modeled traits.

**Supplementary Figure S4.** QTL mapping of ΦPSII at different timepoints within the induction (light) and relaxation (phases).

**Supplementary Figure S5.** Overview of genes identified within all quantitative trait loci (QTL) intervals with previously demonstrated roles in photosynthesis.

**Supplementary Figure S6.** Non-photochemical quenching (NPQ) values during induction and relaxation for Arabidopsis mutants of genes orthologous to those associated with the main effectQTL on chromosome 1.

**Supplementary Figure S7.** Non-photochemical quenching (NPQ) values during induction and relaxation for Arabidopsis mutants of genes orthologous to those associated with the main effectQTL on chromosome 5.

**Supplementary Figure S8.** Phylogenetic tree reconstruction for ZmKEA1, −2, −3 and their Arabidopsis orthologs.

**Supplementary Figure S9.** Maximum efficiency of photosystem II (*F*_v_/*F*_m_) for Arabidopsis mutants of genes orthologous to those associated with the main effect QTL on chromosome 10.

**Supplementary Figure S10.** Analyses of Arabidopsis mutants of genes with high sequence similarity to the *Zm0001d048314* protein kinase.

**Supplementary Figure S11.** Summary of results fast rise fluorescence kinetics of F7 and B73.

**Supplementary Figure S12.** Summary of F7 pedigree analyses.

**Supplementary Figure S13.** Principal Components Analysis of the whole genotyped population (*N* = 552) based on SPET genotyping SNPs (*N* = 74,706).

**Supplementary Table S1.** Predicted means for all main measured and modeled traits.

**Supplementary Table S2.** Predicted means for NPQ and ΦPSII at each measured timepoint.

**Supplementary Table S3.** Associations between all pair-wise trait interactions for predicted means derived from the 2021, 2022, and joint-year linear mixed effects model.

**Supplementary Table S4.** Genes underlying all identified QTL.

**Supplementary Table S5.** Genes where expression variation is associated with QTL haplotype effects.

**Supplementary Table S6.** Top SNP associations within QTL confidence intervals (±250 kbp).

**Supplementary Table S7.** BLAST results for *Zm0001d048314*.

**Supplementary Table S8.**  *CCD1* copy number for the founder accessions of the MAGIC population.

**Supplementary Table S9.**CNVs identified within the QTL regions of the joint model.

**Supplementary Table S10.** Allelic variants within the main effect chromosome 10 QTL that are unique to F7.

**Supplementary Table S11.** TFBS profiles of the founders haplotypes at the 4 private variants of F7 identified in the coding region of CP24 ± 1,000 bps.

**Supplementary Table S12.** Genes underlying QTL in this present study and identified as candidate genes in Sahay et al. (2023).

**Supplementary Table S13.** Commonality between genes within the QTL intervals identified across the 2 experimental years in this present study and the genes identified as candidate genes across the experimental years in Sahay et al. (2023).

**Supplementary Table S14.** Summary of F7 pedigree analyses.

**Supplementary Table S15.** Agronomic inputs during the 2021 and 2022 field seasons.

**Supplementary Table S16.** Primers used for verifying Arabidopsis mutants.

**Supplementary Table S17.** Summary of *t*-tests used for statistical analyses of Arabidopsis mutant experiments.

**Supplementary Data Set 1.** FASTA file used for alignments of ZmKEA1, −2, −3.

**Supplementary Data Set 2.** Newick file used for phylogenetic tree reconstruction of ZmKEA1, −2, −3.

**Supplementary Video S1.** QTL for NPQ during the light phase of the experimental protocol using predicted means derived from the joint-year mixed effects model.

**Supplementary Video S2.** QTL for ΦPSII during the light phase of the experimental protocol using predicted means derived from the joint-year mixed effects model.

**Supplementary Video S3.** QTL for NPQ during the dark phase of the experimental protocol using predicted means derived from the joint-year mixed effects model.

**Supplementary Video S4.** QTL for ΦPSII during the dark phase of the experimental protocol using predicted means derived from the joint-year mixed effects model.

## Dive Curated Terms

The following phenotypic, genotypic, and functional terms are of significance to the work described in this paper:

ADH1 Gramene: AT1G77120
ADH1 Araport: AT1G77120

ENA Gramene: AT3G17668
ENA Araport: AT3G17668

RGP1 Gramene: AT3G02230
RGP1 Araport: AT3G02230

CCD1 Gramene: AT3G63520
CCD1 Araport: AT3G63520

ABI4 Gramene: AT2G40220
ABI4 Araport: AT2G40220

KEA3 Gramene: AT4G04850
KEA3 Araport: AT4G04850

CDC48 Gramene: AT3G09840
CDC48 Araport: AT3G09840

kea1 Gramene: AT1G01790
kea1 Araport: AT1G01790

TFBS Gramene: Transcription Factor Binding Sites (TFBSs)
TFBS Araport: Transcription Factor Binding Sites (TFBSs)
