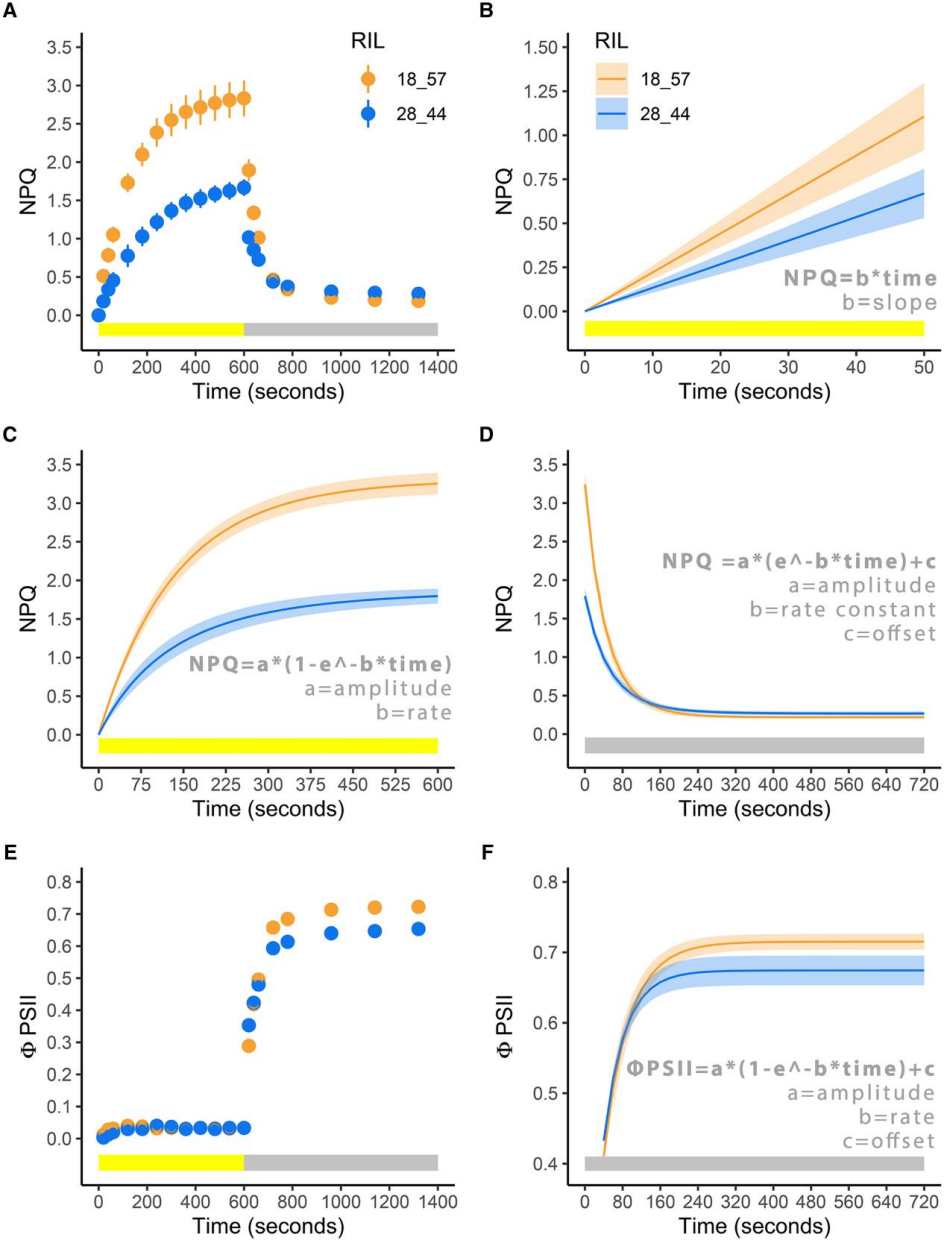# Correction to: A deficient CP24 allele defines variation for dynamic nonphotochemical quenching and photosystem II efficiency in maize

**DOI:** 10.1093/plcell/koaf158

**Published:** 2025-06-24

This is a **correction** to:

John N Ferguson, Leonardo Caproni, Julia Walter, Katie Shaw, Lucia Arce-Cubas, Alice Baines, Min Soe Thein, Svenja Mager, Georgia Taylor, Lee Cackett, Jyotirmaya Mathan, Richard L Vath, Leo Martin, Bernard Genty, Mario Enrico Pè, Tracy Lawson, Matteo Dell’Acqua, Johannes Kromdijk, A deficient CP24 allele defines variation for dynamic nonphotochemical quenching and photosystem II efficiency in maize, *The Plant Cell*, Volume 37, Issue 4, April 2025, koaf063, https://doi.org/10.1093/plcell/koaf063

Figure 1 illustrates the method of NPQ and ΦPSII parameter estimation by showing two example recombinant inbred lines with contrasting properties. It has come to our attention that the example plots of ΦPSII shown in the original Figures 1E and F were erroneously showing Fv’/Fm’ instead of ΦPSII. The original and corrected version of both panels are provided below. This was an inadvertent mistake that occurred during the assembly of Figure 1 and unfortunately was only discovered after approving the proofs. The original figure legend remains unchanged and the correction does not affect the interpretation or conclusions of the study. The authors apologize for this mistake.

Original Figure 1E and F:

**Figure:**
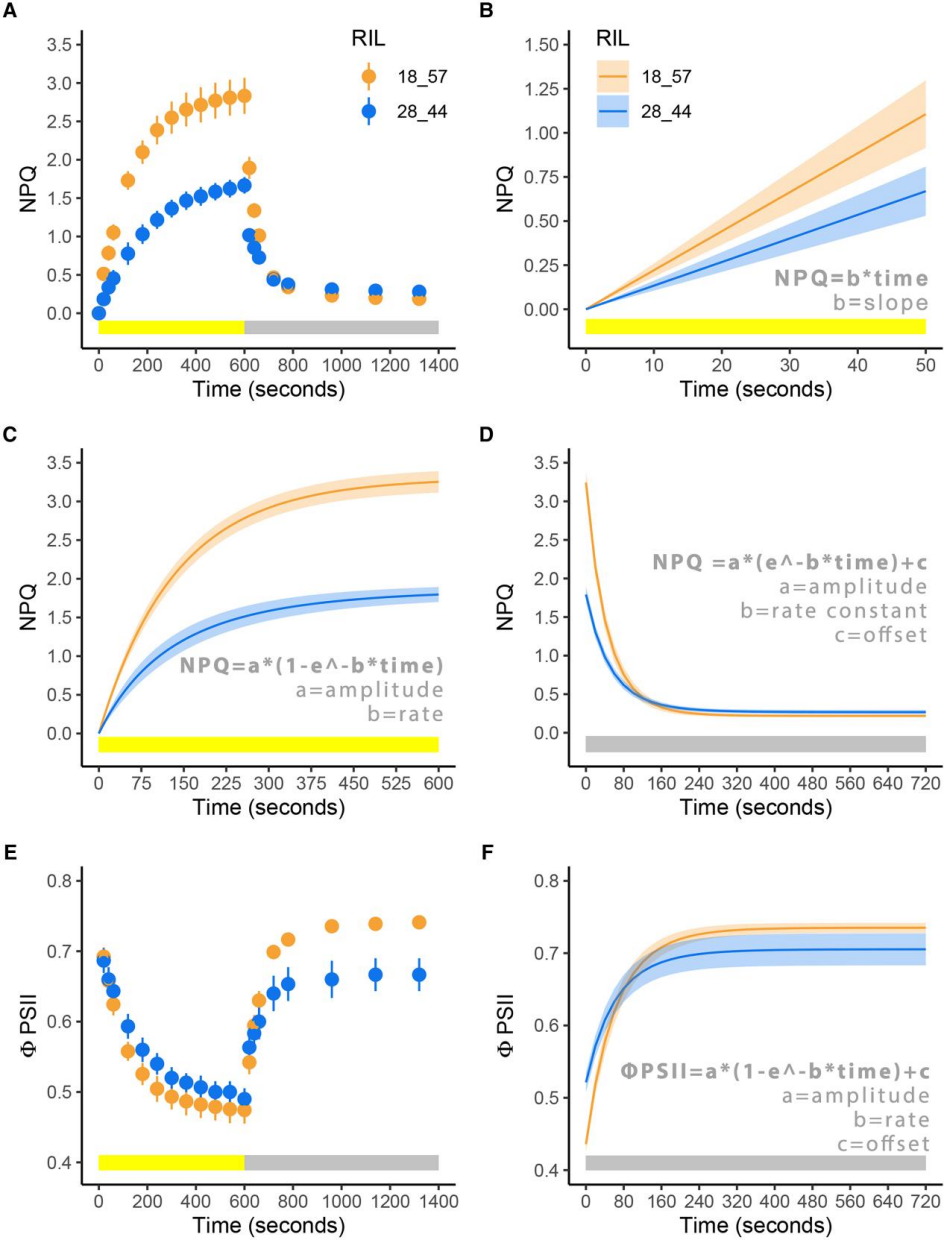


Corrected Figure 1E and F:

**Figure:**