# Supplementary material for: A deficient CP24 allele defines variation for dynamic nonphotochemical quenching and photosystem II efficiency in maize
Source: Plant Cell. 2025 Mar 25;37(4):koaf063. doi: 10.1093/plcell/koaf063 (PMC12018801; doi:10.1093/plcell/koaf063)
Supplement: koaf063_Supplementary_Data [file koaf063_supplementary_data.zip › Supplemental Figures.pdf]

Supplemental Figures

A deficient CP24 allele defines variation for dynamic non-photochemical quenching and photosystem II efficiency in maize

John N. Ferguson[1,2*], Leonardo Caproni[3*], Julia Walter[1], Katie Shaw[1,$], Lucia Arce-Cubas[1], Alice Baines[1], Min Soe Thein[3], Svenja Mager[3], Georgia Taylor[1], Lee Cackett[1], Jyotirmaya Mathan[2], Richard L. Vath[1,&], Leo Martin[4], Bernard Genty[4], Mario Enrico Pè[3], Tracy Lawson[2], Matteo Dell'Acqua[3§], Johannes Kromdijk[1,5§]

*These authors contributed equally

[1]Department of Plant Sciences, University of Cambridge, Cambridge, Cambridgeshire, CB2 3EA, UK

[2]School of Life Sciences, University of Essex, Wivenhoe Park, Colchester, Essex, CO4 3SQ, UK

[3]Institute of Plant Sciences, Scuola Superiore Sant'Anna, Pisa, Italy

[4]Université Aix-Marseille, Commissariat à l'Energie Atomique, Centre National de la Recherche Scientifique, Unité Mixte de Recherche 7265, Institut de Biosciences et Biotechnologies Aix-Marseille, 13108 Saint-Paul-lez- Durance, France

[5]Institute for Genomic Biology, University of Illinois at Urbana-Champaign, Urbana, Illinois, 61801, USA

[$]Present Address: School of Biosciences, University of Sheffield, Sheffield, UK

[&]Present Address: LI-COR Biosciences, Lincoln, NE, USA

[§]Corresponding authors: Matteo Dell'Acqua (m.dellacqua@santannapisa.it); Johannes Kromdijk (jk417@cam.ac.uk)

The authors responsible for distribution of materials integral to the findings presented in this article in accordance with the policy described in the Instructions for Authors (https://academic.oup.com/plcell/pages/General-Instructions) are Matteo Dell'Acqua (m.dellacqua@santannapisa.it) and Johannes Kromdijk (jk417@cam.ac.uk).

Supplemental Figures

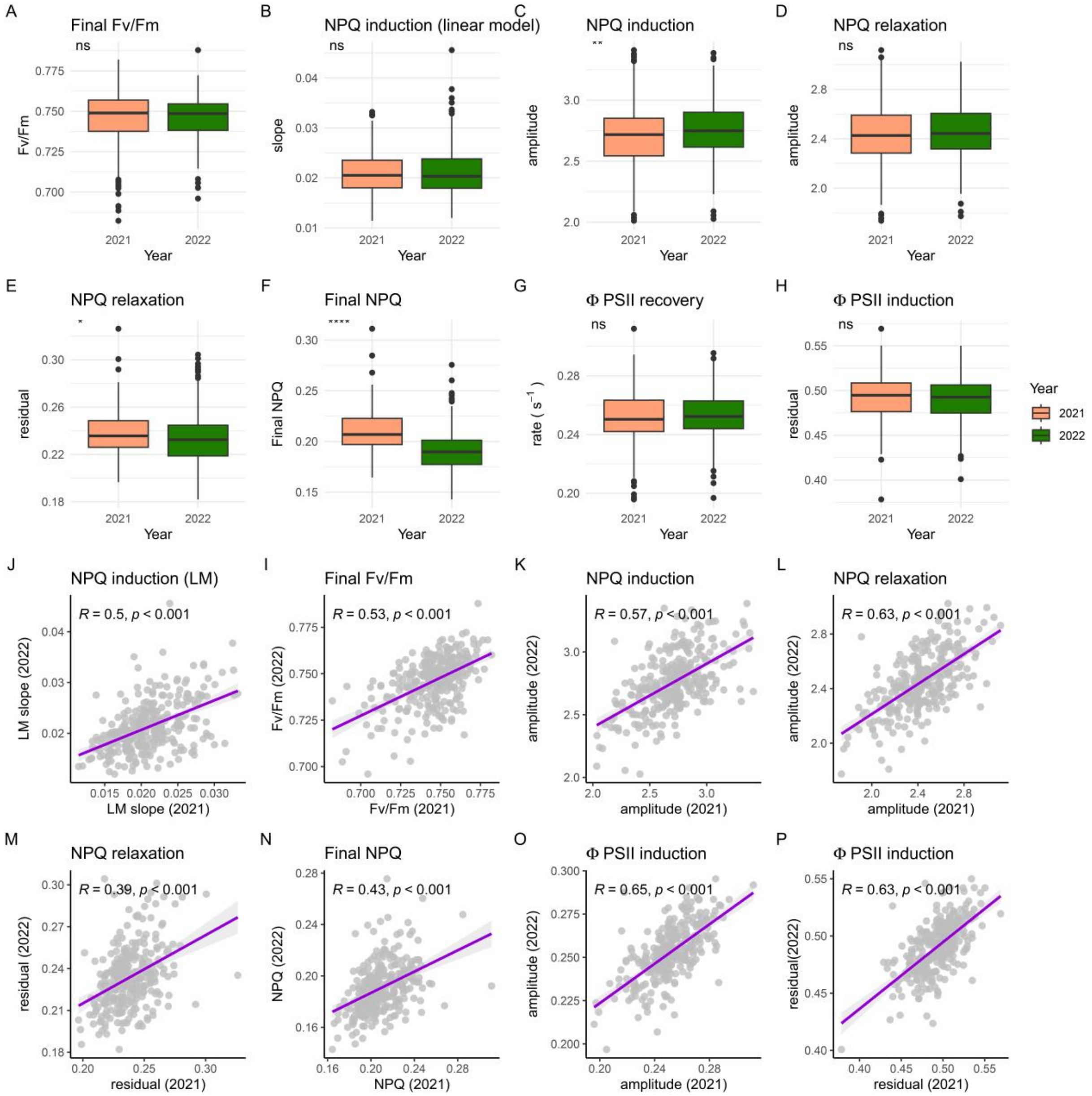

Supplemental Figure S1. Comparison of selected parameters across the two experimental years. (A-H) Boxplots demonstrating population wide variation across both experimental years for final operating efficiency of photosystem II (ΦPSII), non-photochemical quenching (NPQ) induction (slope from linear model), NPQ induction amplitude, NPQ relaxation amplitude, NPQ relaxation residual, Final NPQ, ΦPSII recovery amplitude, ΦPSII recovery residual. The boxes of the boxplots denote the median and interquartile range. The whiskers show the minimum and maximum range, with recombinant inbred lines falling away from that range being shown as individual circles. 2021 N = 316; 2022 N = 312. Differences between 2021 and 2022 were statistically tested via one-way ANOVAs. (I-P) Scatter plots demonstrating associations between the same traits across each experimental year. Correlations between 2021 and 2022 were statistically tested via a linear model, with the associated regression line and standard error being denoted as the line and associated shaded area (N = 301). Significant differences and correlations are denoted at the following levels: * 0.05, ** 0.01, *** 0.001, **** 0.0001.

Supplemental Figures

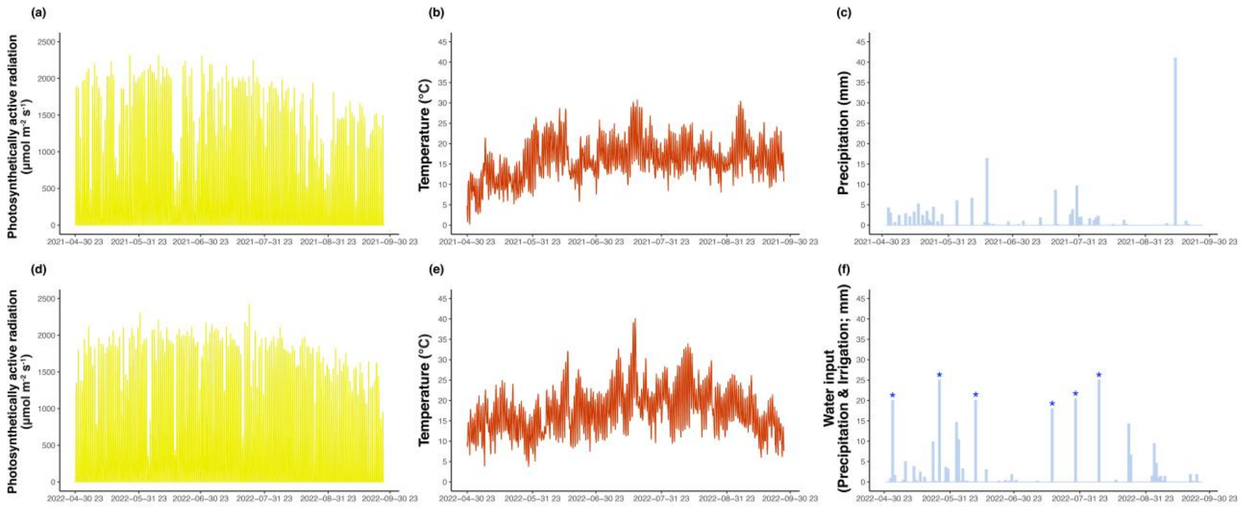

Supplemental Figure S2. Environmental parameters across the two experimental growing seasons. (A) Photosynthetically active radiation (PAR) in 2021. (B) Temperature in 2021. (C) Precipitation in 2021. (D) PAR in 2022. (E) Temperature in 2022. (F) Water inputs (precipitation and irrigation in 2022). Days where irrigation was applied are highlighted with asterisks.

Supplemental Figures

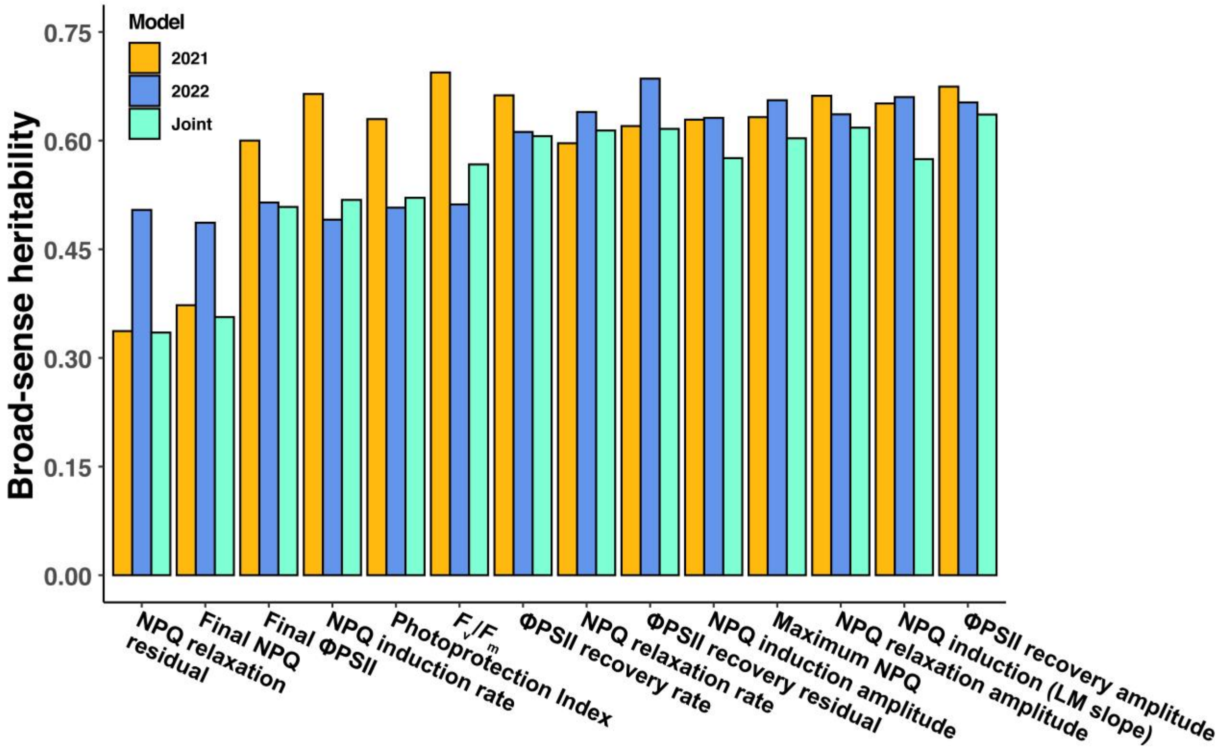

Supplemental Figure S3. Broad-sense heritability for the main measured and modelled traits. Heritabilities are derived from the 2021 (orange), 2022 (blue), and joint-year (mint green) linear mixed effects models.

Supplemental Figures

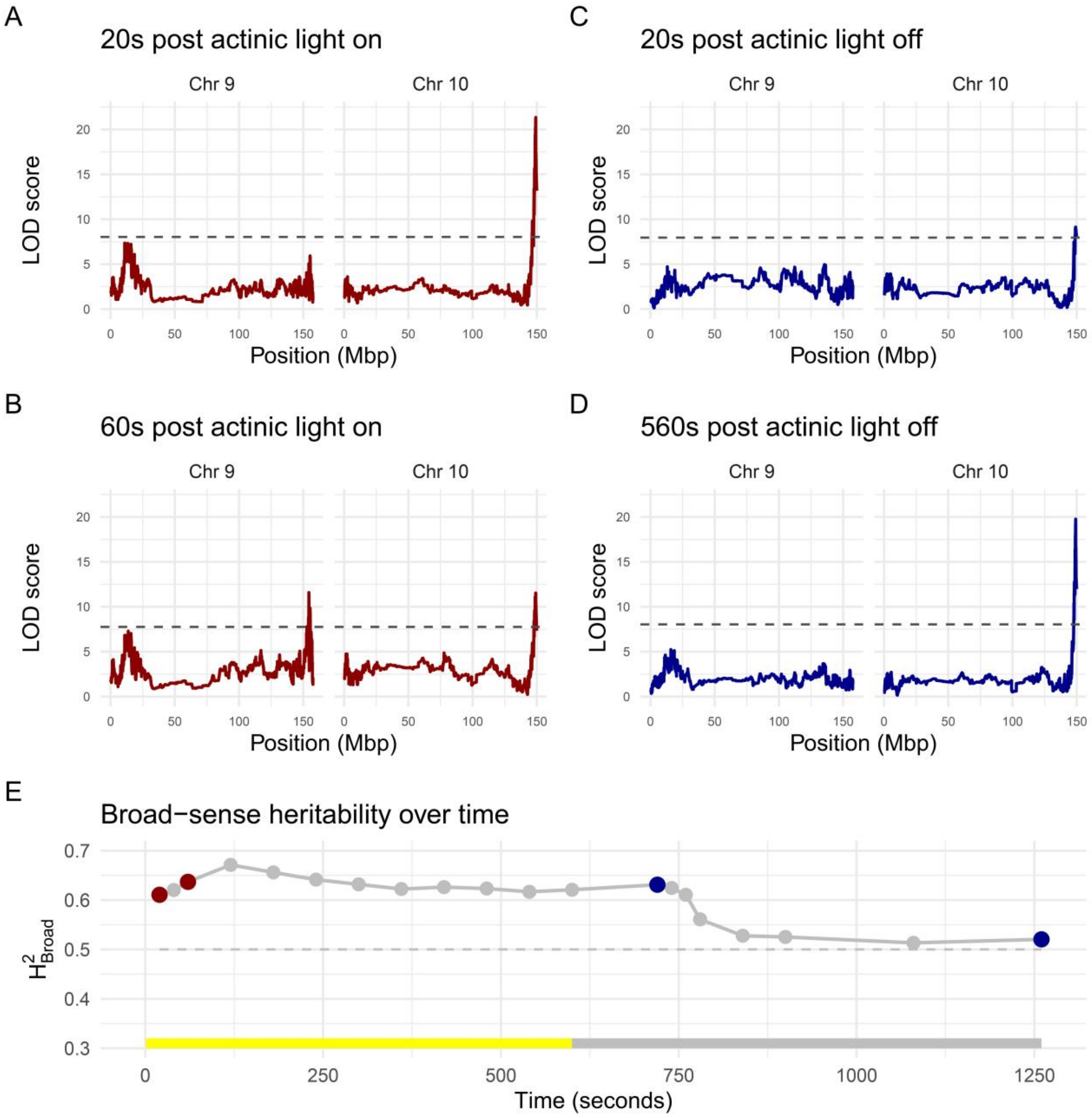

Supplemental Figure S4. QTL mapping of ΦPSII at different timepoints within the induction (light) and relaxation (phases). Mapping is performed using the predicted means derived from the joint-year linear mixed effect models. (A) QTL for ΦPSII 20 seconds after the actinic light is switched on. (B) QTL for ΦPSII 60 seconds after the actinic light is switched on. (C) QTL for ΦPSII 20 seconds after the actinic light is switched off. (D) QTL for NPQ 560 seconds after the actinic light is switched off. (E) Broad-sense heritability (H^2_B) for ΦPSII over time.

Supplemental Figures

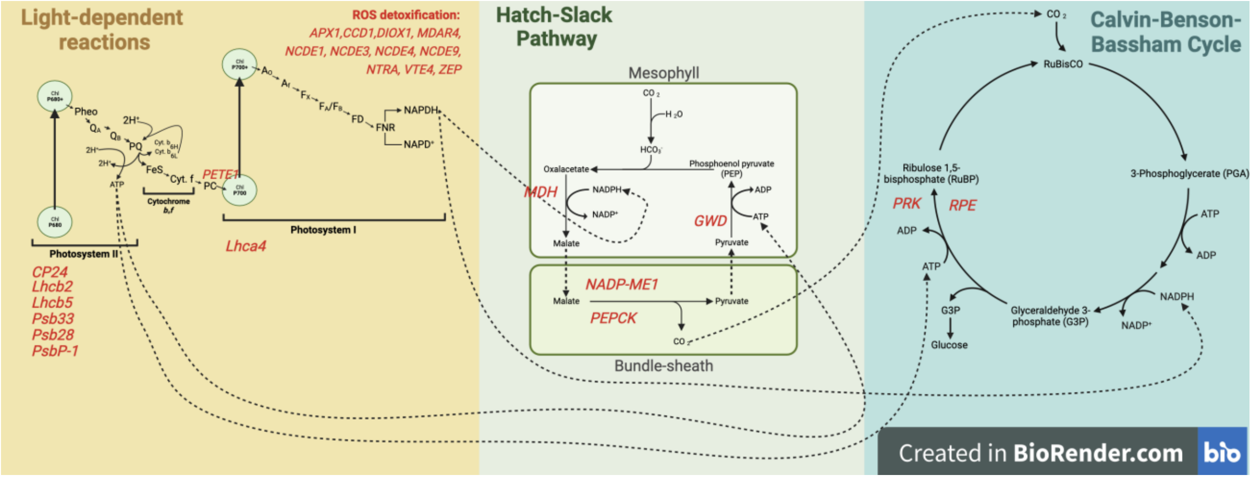

Supplemental Figure S5. Overview of genes identified within all quantitative trait loci (QTL) intervals with previously demonstrated roles in photosynthesis. Genes involved in the light dependent photosynthetic reactions, detoxification of reactive oxygen species (ROS), the Hatch-Slack Pathway (C4 photosynthesis), and the Calvin-Benson-Bassham Cycle (C3 photosynthesis) are highlighted and positioned approximately within the sites of these pathways for which they contribute to associated processes.

Supplemental Figures

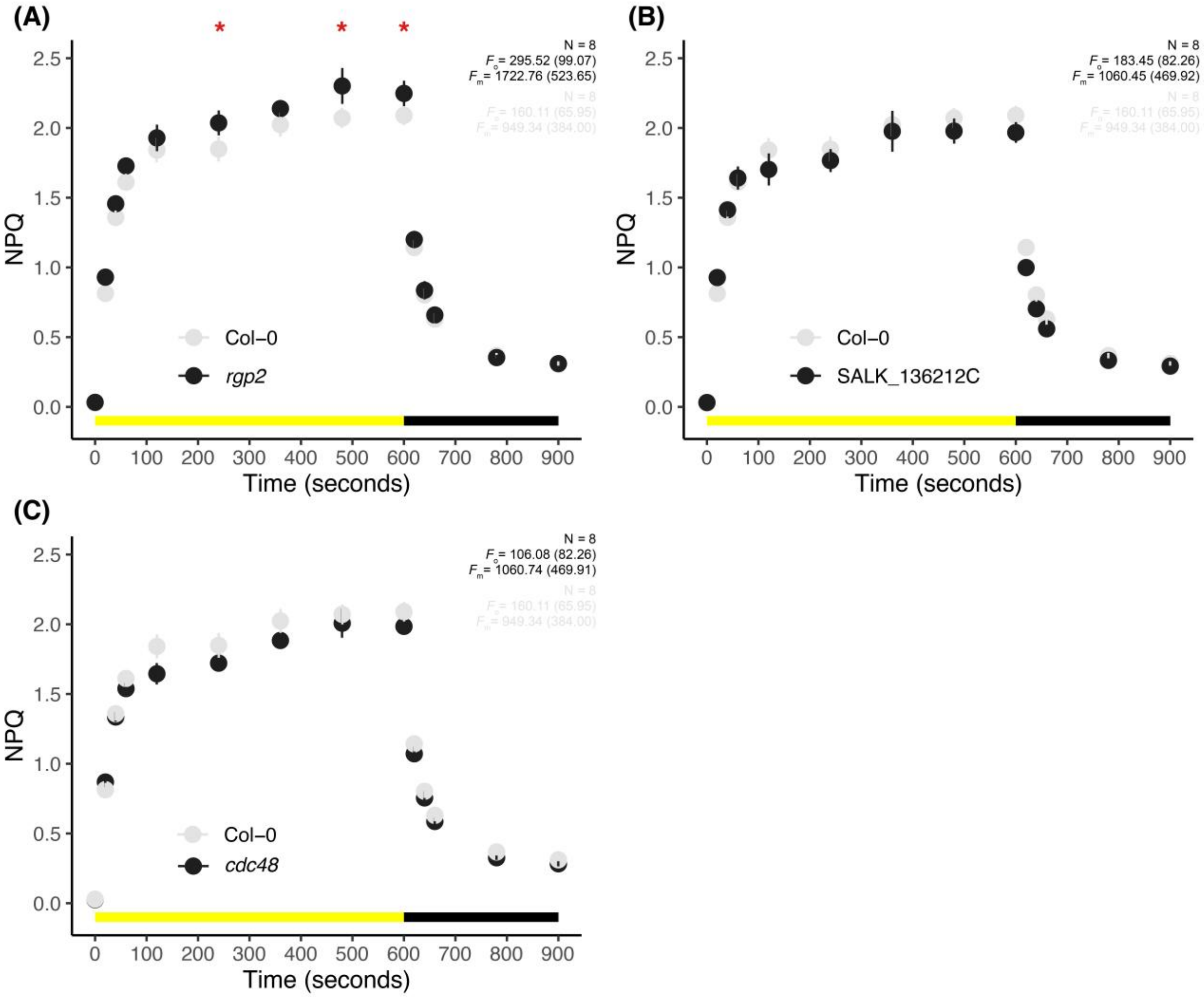

Supplemental Figure S6. Non-photochemical quenching (NPQ) values during induction and relaxation for Arabidopsis mutants of genes orthologous to those associated with the main effect quantitative trait loci (QTL) on chromosome one. Significant differences between wildtype and mutants were tested via T-test and are indicated with red asterisks (*P*-value < 0.05, N = 8). (A) Col-0 vs REVERSIBLY GLYCOSYLATE POLYPEPTIDE 2 mutant (*rgp2*); (B) Col-0 vs AT5G02430 mutant (SALK_136212C); (C) Col-0 vs CDC48 mutant (*cdc48*).

Supplemental Figures

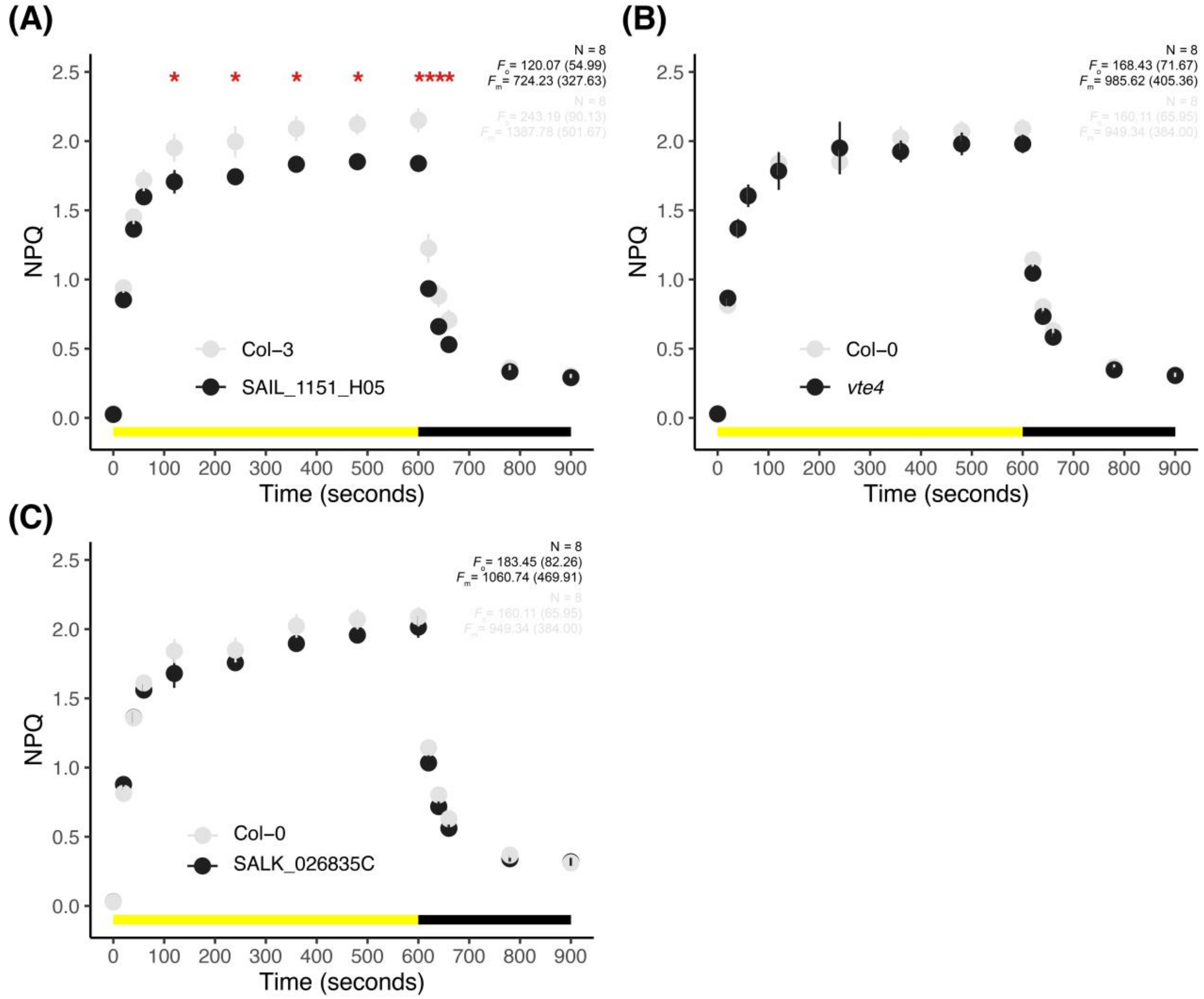

Supplemental Figure S7. Non-photochemical quenching (NPQ) values during induction and relaxation for Arabidopsis mutants of genes orthologous to those associated with the main effect quantitative trait loci (QTL) on chromosome five. Significant differences between wildtype and mutants were tested via T-test and are indicated with red asterisks (*P*-value < 0.05, N = 8). (A) Col-3 vs AT1G76550 mutant (SAIL_1151_H05); (B) Col-0 vs VITAMIN E DEFICIENT 4 mutant (*vte4*); (C) Col-0 vs AT2G17410mutant (SALK_026835C).

Supplemental Figures

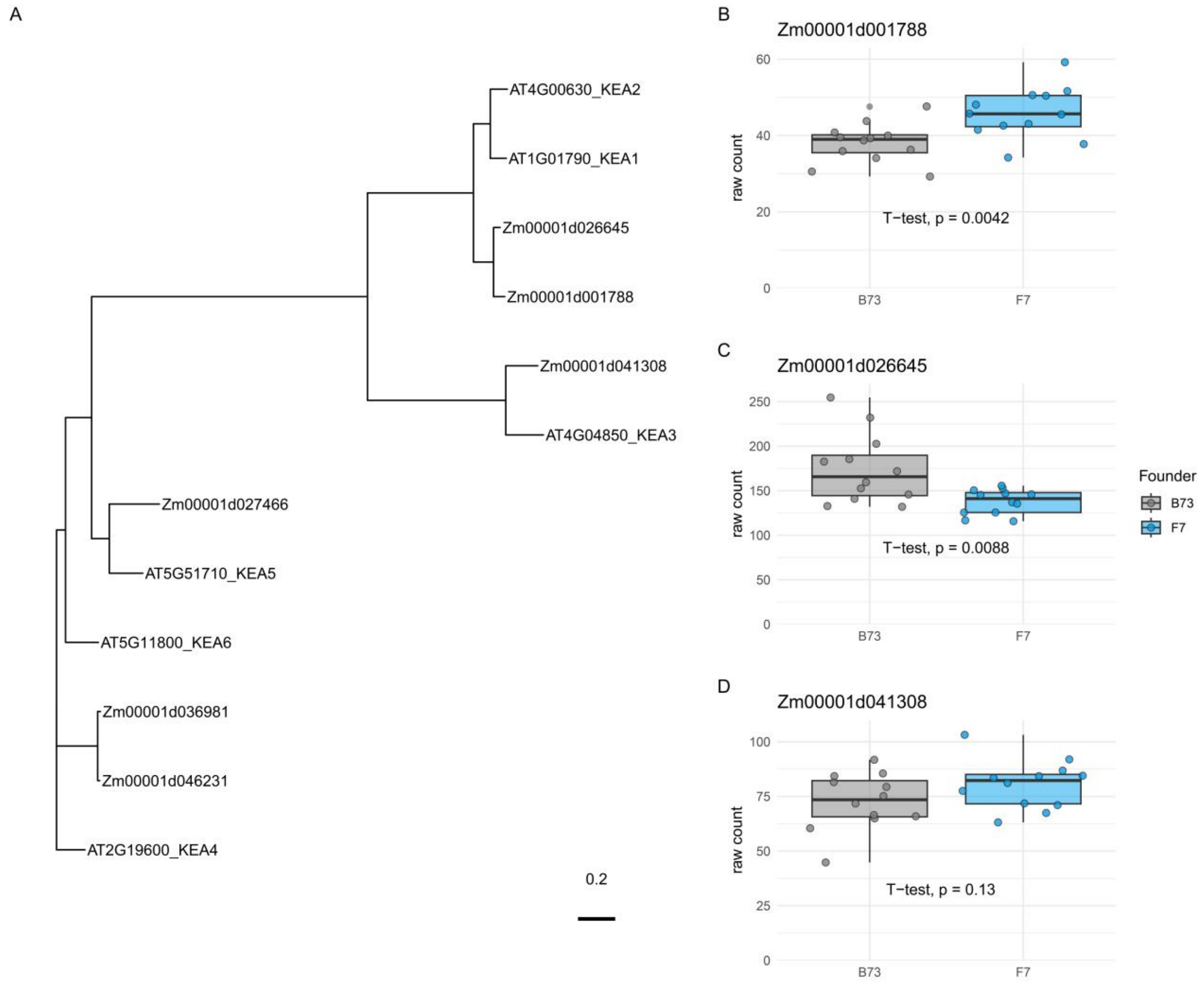

Supplemental Figure S8. Phylogenetic tree reconstruction for ZmKEA1, -2, -3 and their Arabidopsis orthologs. (A) Maize sequences were obtained from multiple sequence alignment (using MUSCLE) of the protein sequence Zm00001d026645 against the maize reference genome hosted on PLAZA monocot (V5). The phylogenetic tree was constructed with FastTree version 2.1. The branch length is proportional to evolutionary distance displayed as rate of amino acid substitutions. Difference in expression levels for (B) Zm00001d001788 (C) Zm00001d026645 and (D) Zm00001d041308 between B73 and F7 from 3'mRNAseq data by Cackett et al. (2023) (N = 12). The boxes of the boxplots denote the median and interquartile range. The whiskers show the minimum and maximum range. The raw data of each biological repeat is overlaid as circles.

Supplemental Figures

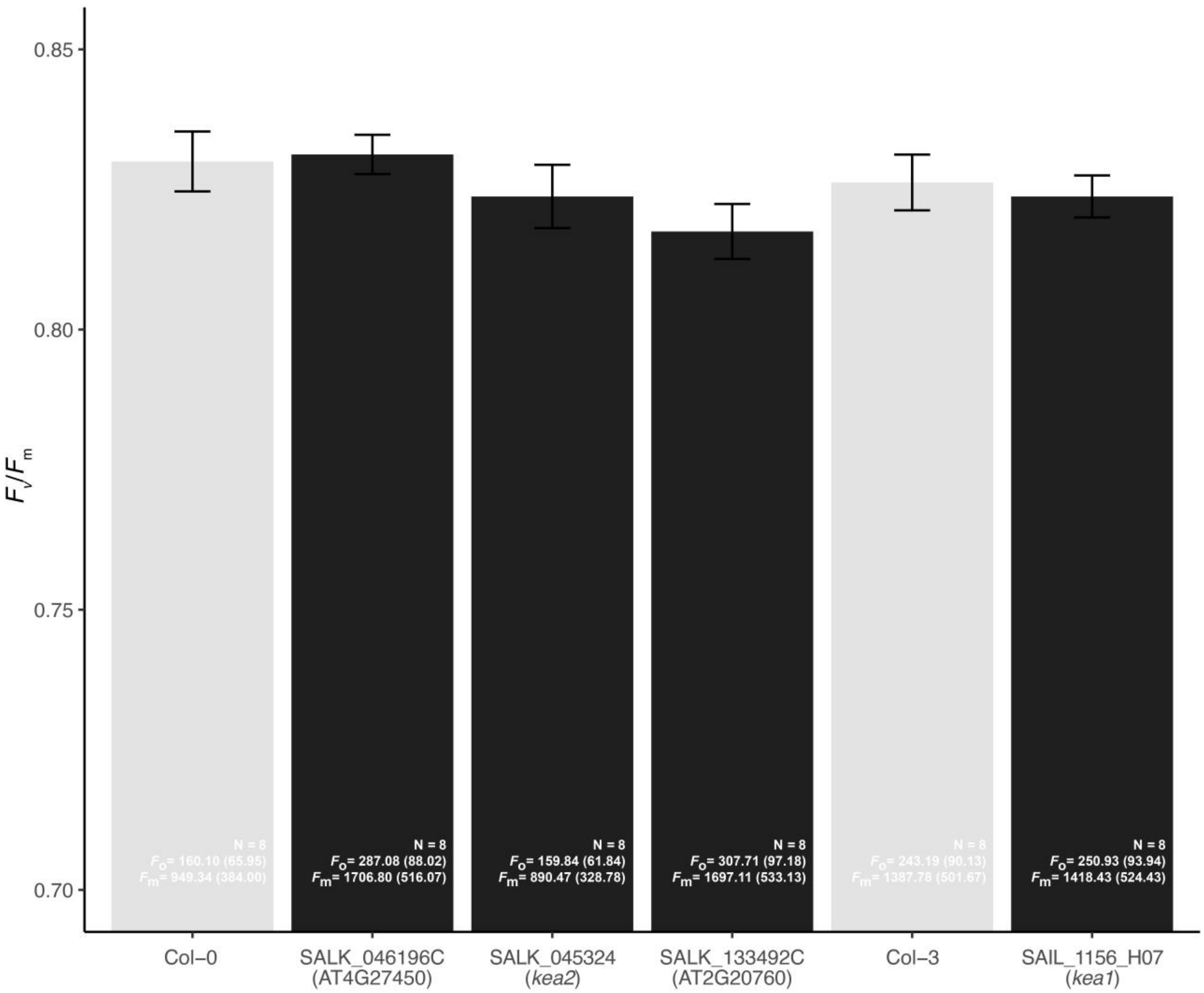

Supplemental Figure S9. Maximum efficiency of photosystem II (F_v/F_m) for Arabidopsis mutants of genes orthologous to those associated with the main effect QTL on chromosome ten. The first three mutants are in the Col-0 genomic background and the final mutant is in the Col-3 background. No significant differences (tested via T-test, N = 8) between mutants and associated wildtypes were detected. Error bars represent the standard error of the mean.

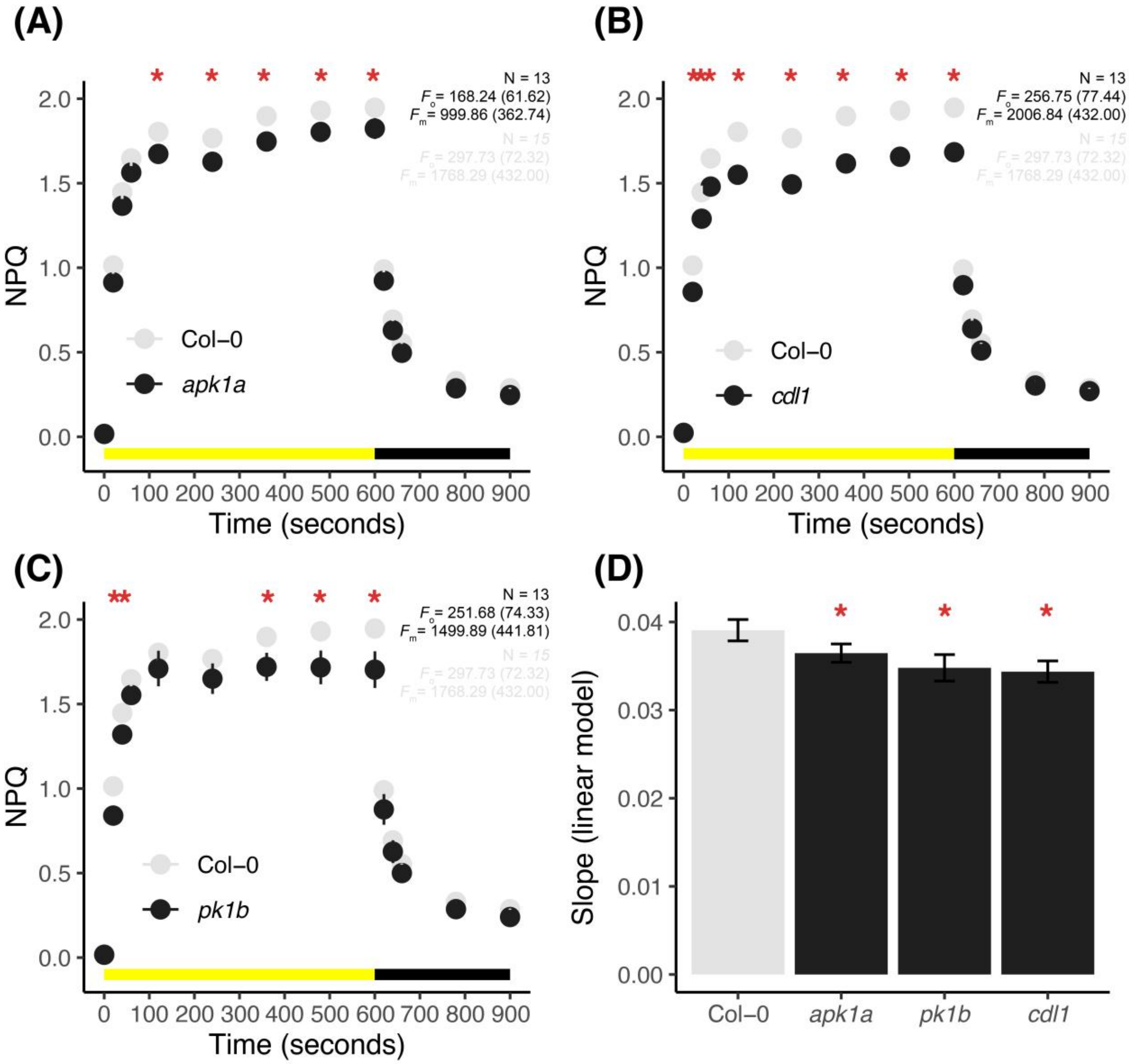

Supplemental Figure S10. Analyses of Arabidopsis mutants of genes with high sequence similarity to the *Zm0001d048314* protein kinase. (A-C) Non-photochemical quenching (NPQ) values during induction and relaxation for mutants from the protein kinase gene family demonstrating sequence similarity to the gene of interest interested identified via the local GWAS approach for the main effect QTL on chromosome nine. (D) Rate of NPQ induction (linear model slope) for these mutants. Significant differences between mutants and wildtype Col-0 were tested via T-test and associated differences are indicated by red asterisks ($P < 0.05$, N = 13-15). Error bars represent the standard error of the mean.

Supplemental Figures

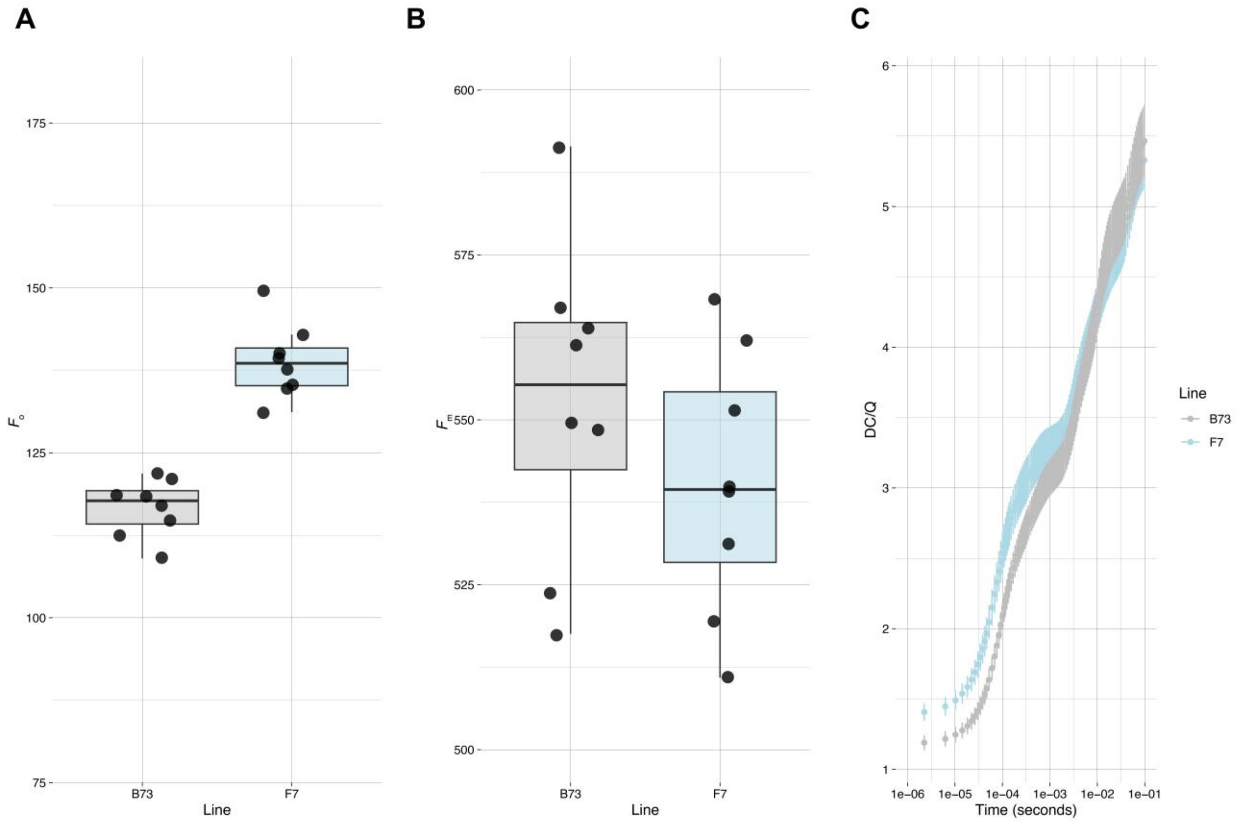

Supplemental Figure S11. Summary of results fast rise fluorescence kinetics of F7 and B73. (A) Differences in F_o. (B) Difference in F_m. (C) Differences in OJIP transients. The boxes of the boxplots denote the median and interquartile range The whiskers show the minimum and maximum range, with recombinant inbred lines falling away from that range being shown as individual circles (N = 8).

Supplemental Figures

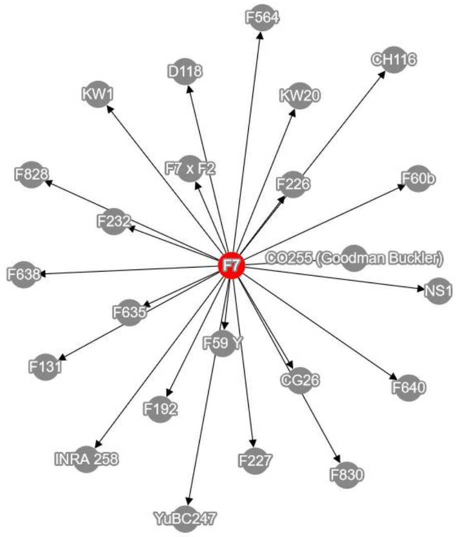

Supplemental Figure S12. Summary of F7 pedigree analyses.

Supplemental Figures

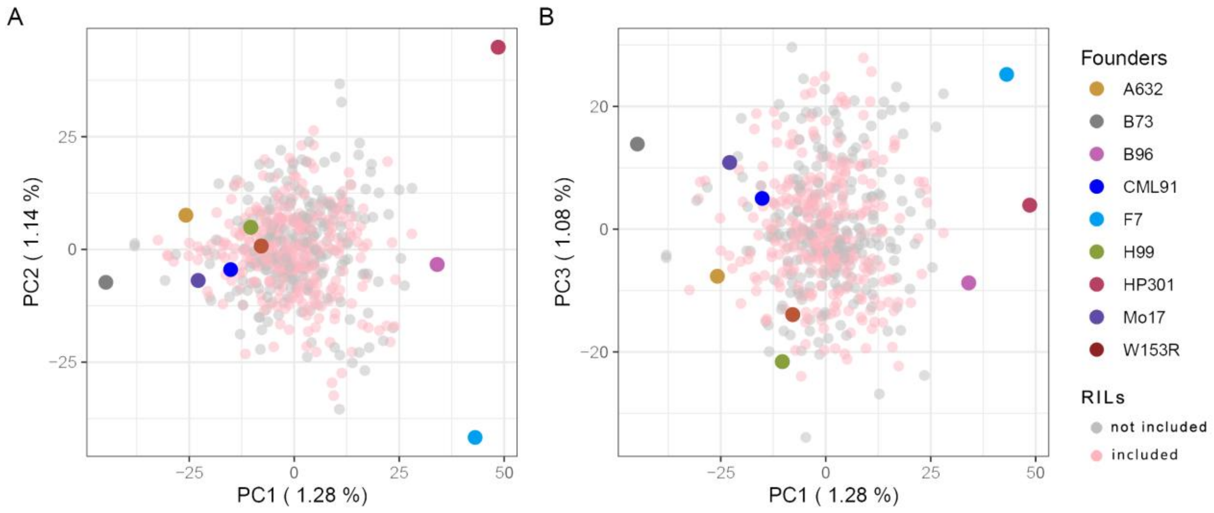

Supplemental Figure S13. Principal Components Analysis of the whole genotyped population (552) based on SPET genotyping SNPs (74,706): (A) PC1 vs PC2 (B) PC1 vs PC3. While founders are highlighted with different colors and larger dots, RILs include in this study are highlighted in light pink.
