# Supplementary material for: A deficient CP24 allele defines variation for dynamic nonphotochemical quenching and photosystem II efficiency in maize
Source: Plant Cell. 2025 Mar 25;37(4):koaf063. doi: 10.1093/plcell/koaf063 (PMC12018801; doi:10.1093/plcell/koaf063)
Supplement: koaf063_Supplementary_Data [file koaf063_supplementary_data.zip › Supplementary Video Legends.docx]

Supplemental Video 1. QTL for NPQ during the light phase of the experimental protocol using predicted means derived from the joint-year mixed effects model.

Supplemental Video 2. QTL for ΦPSII during the light phase of the experimental protocol using predicted means derived from the joint-year mixed effects model.

Supplemental Video 3. QTL for NPQ during the dark phase of the experimental protocol using predicted means derived from the joint-year mixed effects model.

Supplemental Video 4. QTL for ΦPSII during the dark phase of the experimental protocol using predicted means derived from the joint-year mixed effects model.
