# Supplementary figures and images for: A deficient CP24 allele defines variation for dynamic nonphotochemical quenching and photosystem II efficiency in maize
Source: Plant Cell. 2025 Mar 25;37(4):koaf063. doi: 10.1093/plcell/koaf063 (PMC12018801; doi:10.1093/plcell/koaf063)

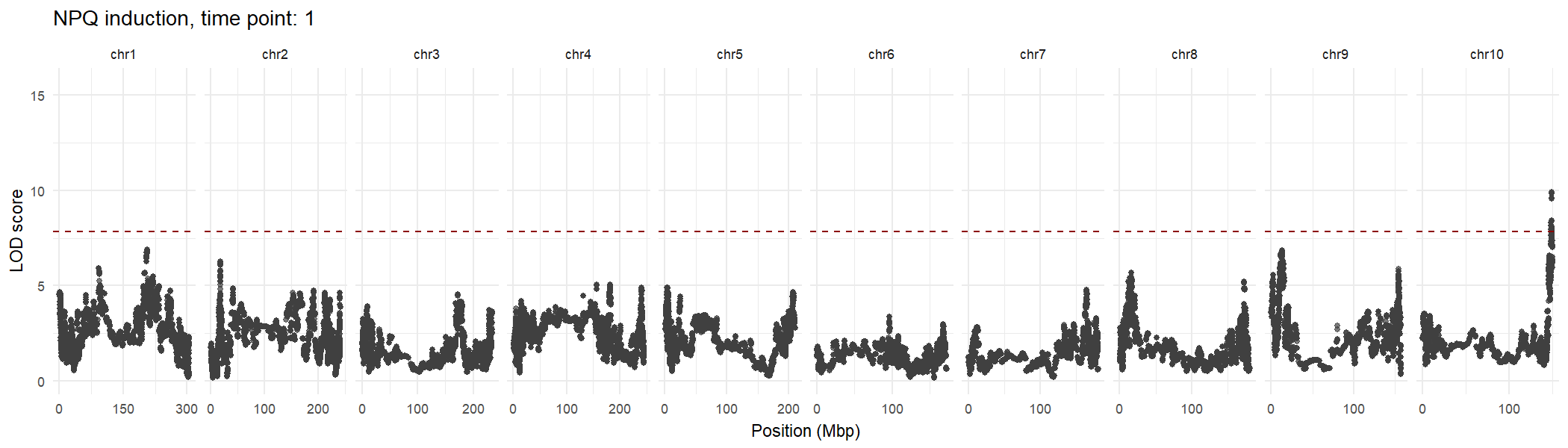

Supplement: koaf063_Supplementary_Data [file koaf063_supplementary_data.zip › Supplemental video 1 - NPQ_induction.gif]

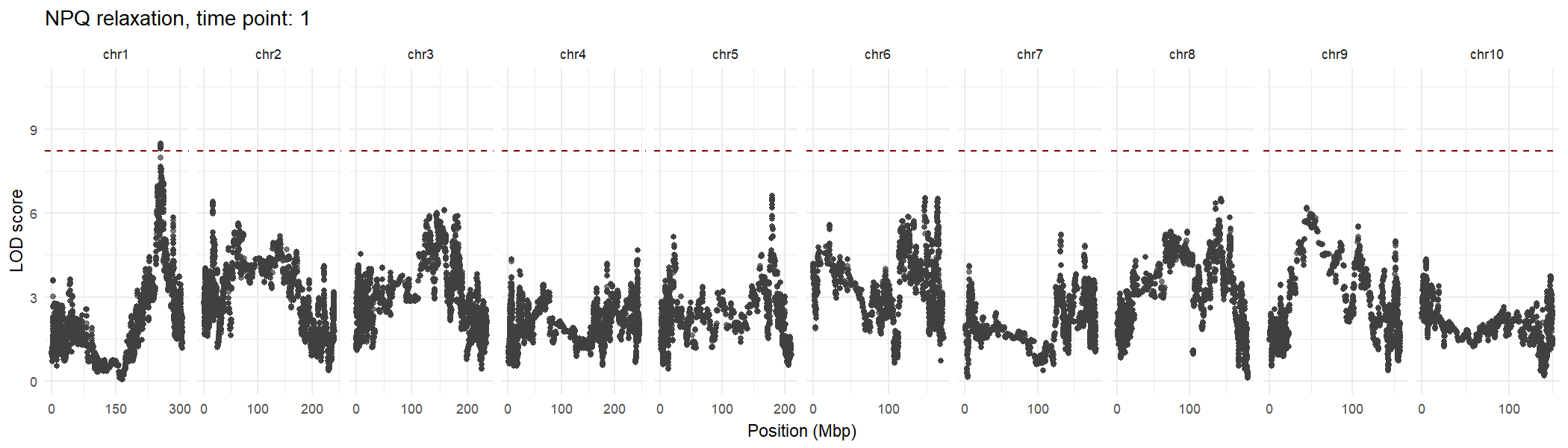

Supplement: koaf063_Supplementary_Data [file koaf063_supplementary_data.zip › Supplemental video 2 - NPQ_relaxation.gif]

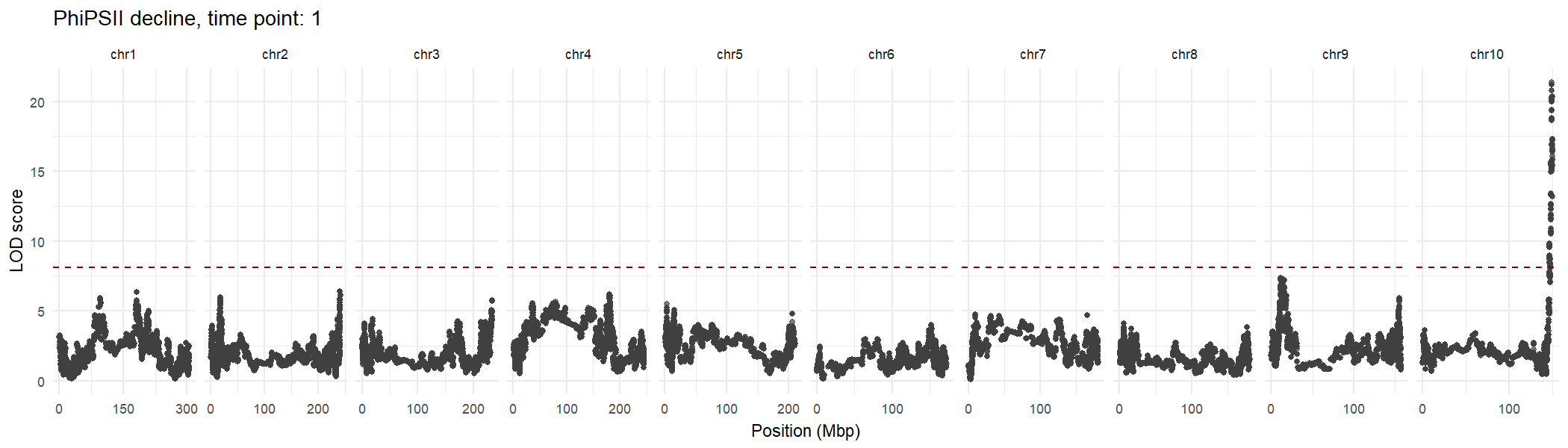

Supplement: koaf063_Supplementary_Data [file koaf063_supplementary_data.zip › Supplemental video 3 - PhiPSII_decline.gif]

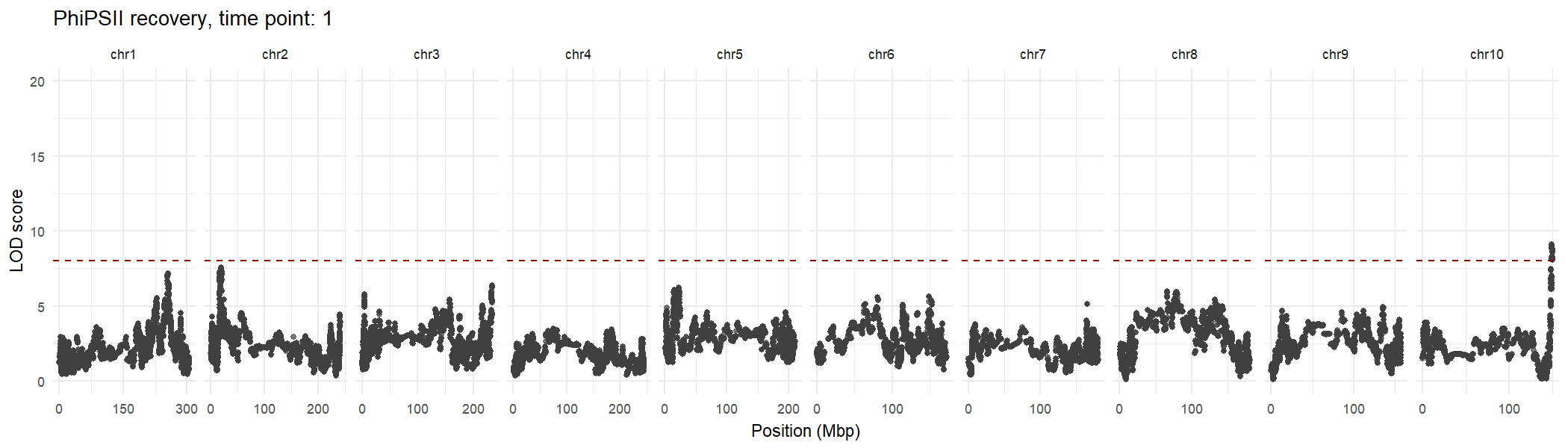

Supplement: koaf063_Supplementary_Data [file koaf063_supplementary_data.zip › Supplemental video 4 - PhiPSII_recovery.gif]
